# A role of gut microbiota metabolites in HLA-E and NKG2 blockage immunotherapy against tumors: new insights for clinical application

**DOI:** 10.3389/fimmu.2024.1331518

**Published:** 2024-08-20

**Authors:** Wenyue Cheng, Ningning Zhu, Juanjuan Wang, Rongcun Yang

**Affiliations:** ^1^ Translational Medicine Institute, Affiliated Tianjin Union Medical Center of Nankai University, Nankai University, Tianjin, China; ^2^ Department of Immunology, Nankai University School of Medicine, Nankai University, Tianjin, China; ^3^ State Key Laboratory of Medicinal Chemical Biology, Nankai University, Tianjin, China

**Keywords:** gut microbiota, NKG2A, HLA-E, tumor, immune cells

## Abstract

One of major breakthroughs in immunotherapy against tumor is from blocking immune checkpoint molecules on tumor and reactive T cells. The development of CTLA-4 and PD-1 blockage antibodies has triggered to search for additional effective therapeutic strategies. This causes recent findings that blocking the interaction of checkpoint molecule NKG2A in NK and CD8 T cells with HLA-E in tumors is effective in defensing tumors. Interestingly, gut microbiota also affects this immune checkpoint immunotherapy against tumor. Gut microbiota such as bacteria can contribute to the regulation of host immune response and homeostasis. They not only promote the differentiation and function of immunosuppressive cells but also the inflammatory cells through the metabolites such as tryptophan (Trp) and bile acid (BA) metabolites as well as short chain fatty acids (SCFAs). These gut microbiota metabolites (GMMs) educated immune cells can affect the differentiation and function of effective CD8 and NK cells. Notably, these metabolites also directly affect the activity of CD8 and NK cells. Furthermore, the expression of CD94/NKG2A in the immune cells and/or their ligand HLA-E in the tumor cells is also regulated by gut microbiota associated immune factors. These findings offer new insights for the clinical application of gut microbiota in precise and/or personalized treatments of tumors. In this review, we will discuss the impacts of GMMs and GMM educated immune cells on the activity of effective CD8 and NK cells and the expression of CD94/NKG2A in immune cells and/or their ligand HLA-E in tumor cells.

## 1 Introduction

One of the major breakthroughs in immunotherapy against tumors is from blocking immune checkpoint molecules on tumor and reactive T cells. The development of CTLA (cytotoxic T lymphocyte associate protein)-4 and PD (programmed cell death protein)-1 immunosuppressive antibodies has triggered to look for additional effective therapeutic strategies against tumors.

Indeed, beyond CTLA-4 and PD-1, other immune check point molecules such as NKG2A, TIM (T cell immunoglobulin domain and mucin domain)-3, LAG (lymphocyte activation gene)-3, and TIGIT (T-cell immune-receptor with Ig and ITIM domains), which are expressed in CD8 and natural killer (NK) cells can also be referred to tumor resistance to immune cells (1, 2). A key mechanism of tumor resistance to immune cells is mediated by expression of peptide-loaded HLA (human leukocyte antigen)-E in tumor cells, which suppresses NK and CD8 cell activity via ligation of the NK inhibitory receptor CD94/NKG2A. The large number of human solid tumors such as colon, lung, pancreas, stomach, liver, head and neck carcinomas, in which the overexpression of NKG2A receptor and/or their ligand HLA-E has been often associated with a poor prognosis (3–5). Recently, several studies have examined targeting of the immune checkpoint NKG2A through either combined with a tumor-targeting antibody or with a tumor-specific vaccine, and demonstrated that blocking the interaction of NKG2A on both NK cells and CD8^+^ T cells and HLA-E in the tumor cells is effective in defensing tumor (6) through enhancing effective functions of both NK and CD8^+^ T cells in mice and humans (7–12).

Interestingly, gut microbiota such as bacteria, fungi and viruses can directly and indirectly affect immune responses in HLA-E and NKG2 blockage immunotherapy against tumors. The metabolites from specific microorganisms in gut microbiota such as short chain fatty acid (SCFAs), tryptophan (Trp) and bile acid (BA) metabolites may regulate the differentiation and function of immune cells through genetic, epigenetic and metabolic regulation. These immune cells include regulatory (suppressive) macrophages (rMacs), regulatory dendritic cells (rDCs), myeloid-derived suppressive cells (MDSCs), regulatory T cells (Tregs), regulatory B cells (Breg) and effective or inflammatory cells such as CD4 T helper (Th)1, TH2, TH17, NKT cells, NK cells, neutrophils and innate lymphocytes (ILCs). They express different receptors such as G-protein coupled receptor (GPR)43 for SCFAs, aryl hydrocarbon receptor (AhR) for Trp metabolites, and farnesoid X receptor (FXR), vitamin D receptor (VDR), liver-X-receptor (LXR), pregnane X receptor (PXR), retinoid related orphan receptor (RORγt) and constitutive androstane receptor (CAR) for BA metabolites (13, 14). These GMM educated immunosuppressive and inflammatory cells can influence the functions of effective CD8 and NK cells in HLA-E and NKG2 blockage immunotherapy against tumors such as cytotoxic function. Notably, these metabolites from gut microbiota also directly produce the effects on the function of CD8 and NK cells. Furthermore, the expression of immune checkpoint molecule CD94/NKG2A in the CD8 and NK cells and/or their ligand HLA-E in the tumor cells is also regulated by immune factors such as cytokines. In this review, we will discuss the direct and indirect impacts of GMMs on the activity of effective CD8 and NK cells and on the expression of CD94/NKG2A in the immune cells and/or their ligand HLA-E in the tumor cells.

## 2 HLA-E: NKG2A/CD94 blockage for tumor immunotherapy

### 2.1 HLA-E (H2-T23)

Human leukocyte antigen HLA-E and its mouse ortholog H2-T23 (Qa-1^b^) is lowly expressed on almost all cell surfaces. In tumor cells (hematological as well as solid tumors), HLA-E is frequently overexpressed as compared to their non-transformed counterparts, such as lung, cervix and head/neck carcinoma to avoid killing. In addition, other cells such as Macs, monocytes, and neutrophils also contribute to the expression of HLA-E (11, 15). HLA-E and H2-T23 or Qa-1 displays limited polymorphism. Both HLA-E and H2-T23 are also conserved in the population and present signal peptides of classical MHC class I molecules. Its stabilization at the cell surface is dependent on the availability of peptide ligands and proper function of the antigen processing machinery. HLA-E, which is primarily loaded with VL9 that are derived from signal peptides (SPs) of classical HLA class I allotype, can serve as a ligand for CD94/NKG2A and CD94/NKG2C receptors expressed on NK and T cell subsets. Notably, the expression of H2-T23 (HLA-E) molecules can not only be regulated by genetic factors but also influenced by the availability of conserved lead peptide, peptide transporter TAP and proteolytic enzyme through post-translational regulation, which affects the expression of H2-T23 (HLA-E) molecules. Human cytomegalovirus (HCMV) can produce a nonamer peptides bearing valine at position 1 and leucine at position 9 (VL9) that are loaded onto HLA-E, allowing the infected cells to escape from NK cell lysis through CD94/NKG2A: HLA-E-mediated inhibition. However, HLA-E can also bind peptides from viral, bacterial or stress protein origin (6).

### 2.2 CD94/NKG2A

**Figure 1:**
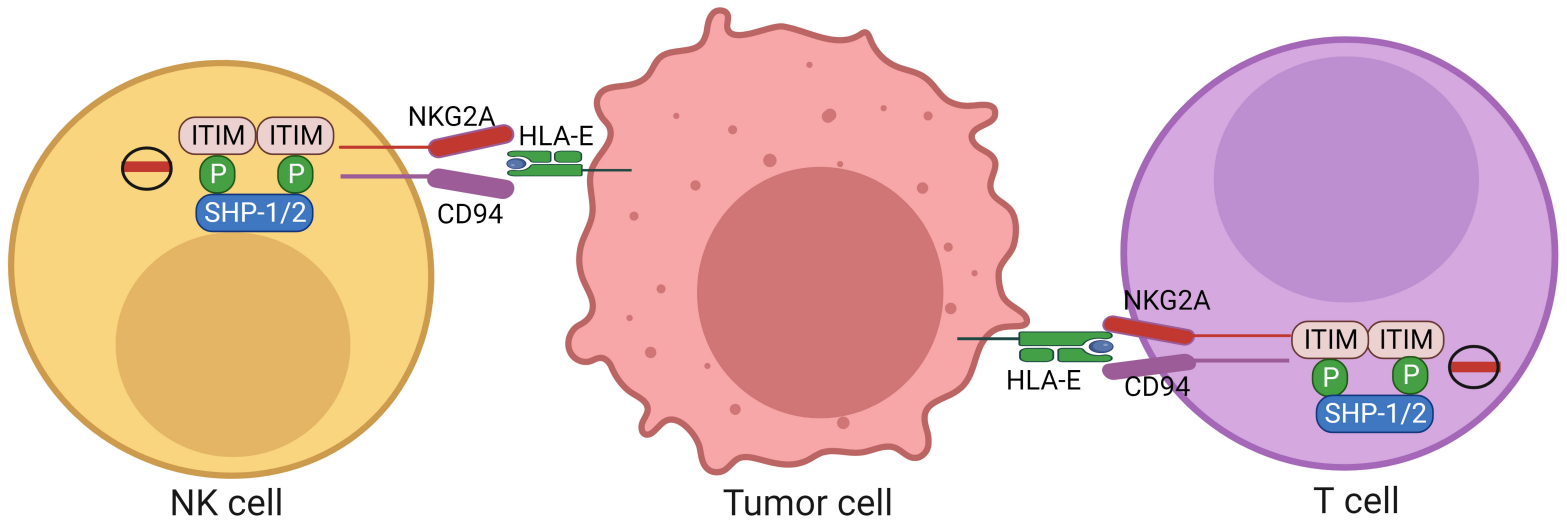
Expression of HLA-E in tumor cells and NKG2A in CD8 and NK cells. HLA-E molecules expressed on the tumor cells can inhibit activity of the CD8 and NK cells through NKG2A expressed on the CD8 and NK cells. The cytoplasmic tail of NKG2A receptor contains two immunoreceptor tyrosine-based inhibition motifs (ITIMs) capable of recruiting both SHP-1 and SHP-2 phosphatases. NK cells, nature killer cells; ITIM, immunoreceptor tyrosine-based inhibition motif; NKG2A, a member of the NKG2 seven receptors; SHP, SH2-containing inositol phosphatase; HLA-E, human leukocyte antigen-E.

NKG2A, a member of the NKG2 seven receptors, namely A, B, C, D, E, F and H, dimerizes with CD94 to form CD94/NKG2A receptor (16). Almost 50% of NK cells in the peripheral blood express CD94/NKG2A. NKG2A can also mark a special CD8^+^ T cell subset with tissue-resident and terminally exhausted features. The frequency of CD8^+^ αβ T cells expressing NKG2A is significantly higher in tumor such as colorectal carcinomas than in paired normal mucosa. Single-cell transcriptomics of human tumor-infiltrating lymphocytes (TILs) show indeed that these receptors are often co-expressed by the CD8 T cell cluster (17, 18). NKG2A belongs to the C-type lectin family of receptors that recognizes non-classical HLA-E molecule. The cytoplasmic tail of NKG2A receptor contains two immune-receptor tyrosine-based inhibition motifs (ITIM) capable of recruiting both SHP (src homology-2 containing protein tyrosine phosphatase)-1 and SHP-2 phosphatases, but not the inositol phosphatase SHIP (SH2-containing inositol phosphatase 1) (19) (**Figure 1**). Both ITIMs can mediate maximal inhibitory signal (20). The partner CD94 lacks ITIMs, and has only seven cytoplasmic amino acids, which has no role in downstream signaling. Other CD94 comprising heterodimers, such as NKG2C, can also bind to HLA-E complexes, but with much lower affinity.

### 2.3 Blocking HLA-E with NKG2A/CD94 for tumor immunotherapy

HLA-E, which is expressed on the tumors, can serve as a ligand for CD94/NKG2A receptor expressed on NK and CD8 cell subsets. Upon binding ligands, CD94/NKG2A receptors deliver signals to suppress NK cell functions. Similar to its function in NK cells, CD94/NKG2A receptors also deliver inhibitory signals to CD8^+^ T cells (6, 8, 11, 21, 22). However, disrupting interaction of CD94/NKG2A with H2-T23 (Qa-1) or HLA-E can activate the cytotoxic activity of CD8^+^ and NK cells (23–25). Clinical trials have demonstrated that monalizumab, a humanized anti-NKG2A antibody, can enhance NK cell activity against various tumor cells and rescue CD8^+^ T cell function in combination with PD-1 blockade (7). A phase II trial of monalizumab with cetuximab in treated squamous cell carcinoma of the head and neck shows a higher response rate to treatment (7). NKG2A^+^ CD8^+^ T cells inhibited by tumors through HLA-E can partly restore upon NKG2A blockade in an HLA-E-dependent manner in the bladder tumors (8). In human pancreatic ductal adenocarcinoma, disruption of this interaction by blockade of NKG2A enhances NK and CD8-mediated tumor cell killing *in vitro*, and also prevents tumor metastasis *in vivo* (26). Thus, immune checkpoint blockages (ICBs) of NKG2A and HLA-E (HLA-E: NKG2A/CD94) is effective in defensing tumors.

Notably, CD8^+^ T cells and NK cells in immune checkpoint blockages (ICBs) of HLA-E: NKG2A/CD94 against tumors can be regulated by other immune cells. For example, CD8^+^ T cells have positive cross-talking with macrophages, CD4^+^ T cells, and dendritic cells (DCs); Whereas there also has a negative cross-talking in CD8^+^ T cells with immunosuppressive cells such as Tregs (27).

## 3 Education of GMMs on the immune cells

**Figure 2:**
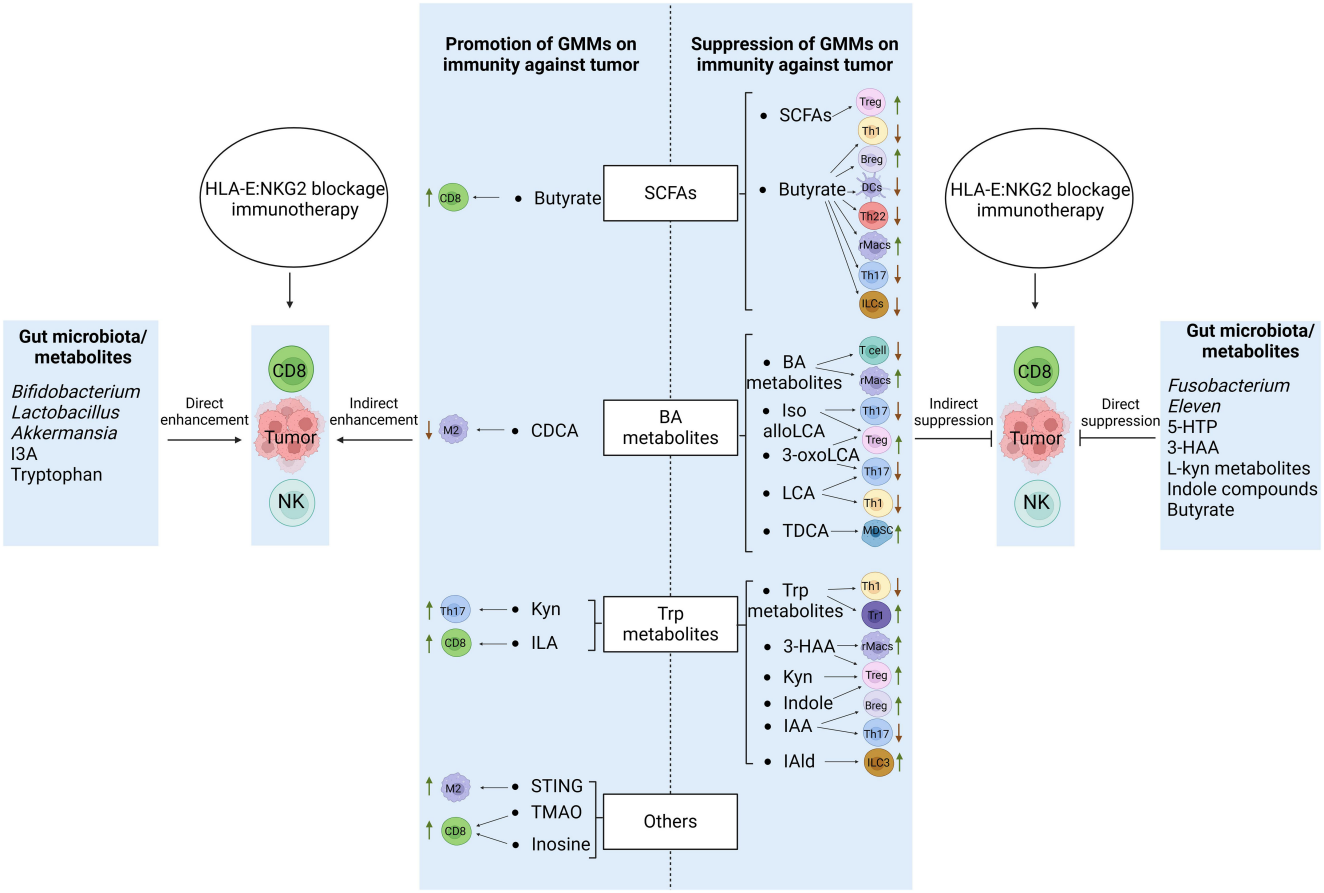
Regulations of GMMs in the CD8 and NK cells of HLA-E: NKG2A blockage immunotherapy against tumors. GMMs affect the function and differentiation of the CD8 and NK cells through not only direct but also indirect roles. Immune regulatory cells such as rMacs, MDSCs, Tregs and Bregs, and immune effective/inflammatory cells such as TH1 and TH17 can be suppressed or promoted by GMMs to positively and negatively regulate the function and differentiation of the CD8 and NK cells. Meanwhile, gut microbiota/GMMs also have directly negative and positive effects on the function and differentiation of CD8 and NK cells. Tregs, regulatory T cells; rMacs, regulatory Macs; Bregs, regulatory B cells; MDSCs, myeloid-derived suppressive cells; DCs, dendritic cells; NK cells, natural killer cells; TH cells, T helper cells; ILCs, innate lymphocytes; SCFAs, short chain fatty acids; CDCA, chenodeoxycholic acid; Kyn, kynurenine; ILA, indole-3-lactic acid; Trp., tryptophan; IPA, indole-3-propionic acid; 3-HAA, 3-hydroxyanthranilic acid; IAA, indole-3-acid-acetic; I3A, indole-3-aldehyde; 5-HTP, 5-hydroxytryptophan;NorUDCA, norursodeoxycholicacid; STING, stimulator of interferon gene; TMAO, trimethylamine oxide.

There have many metabolites derived from gut microbiota. Because of widely effects of Trp metabolites, BA metabolites and SCFAs on the immune cells, especially immunosuppressive cells, we here mainly discuss the roles of these metabolites in regulating immune cells (**Figure 2**).

### 3.1 GMMs

#### 3.1.1 Trp metabolites

Trp metabolism in gut microbiota has been reviewed by us (14) and other multiple papers (28–30). Trp metabolites such as indole (29), indole-3-acid-acetic (IAA) (31–33), indole-3-propionic acid (IPA) (31–33), indoleacrylic acid (IA) (34), indole-3-propionic acid (IPA) (34), indole-3-aldehyde (IAld) (35), skatole (33, 36) and tryptamine can be generated by bacteria in gut microbiota. In addition, gut microbiota bacteria also encode enzymes homologous to those of eukaryotic kynurenine (Kyn) pathway to generate Kyn and downstream metabolites such as 3-hydroxyanthranilic acid (3-HAA) (37).

#### 3.1.2 BA metabolites

Two primary BAs cholic acid (CA) and chenodeoxycholic acid (CDCA) are generated in liver, and then conjugated, deconjugated and transformed into other metabolites in gut microbiota. Gut microbiota bacteria can conjugate glycine to DCA, CDCA or CA *in vitro* (38) or one or more other amino acids such as alanine, arginine and aspartate to CDCA, DCA or CA (38). Four distinct ways, including deconjugation, dehydroxylation, oxidation, and epimerization are used to transform BAs by gut microbiota bacteria in human (39–41). Conjugated BAs are deconjugated in gut bacteria (42–48). After BAs are deconjugated, BAs can be converted into secondary BAs such as DCA and lithocholic acid (LCA). A range of oxo-, epi- and iso-derivatives (49) such as oxo-BA metabolites 3-oxoLCA, 7-oxoCDCA, 12-oxoCA, 7-oxoCA, 12-oxoDCA (50), iso-LCA, 3-oxo-LCA, 3-oxoallo-LCA, isoalloLCA, allo-LCA and 3-ketoLCA can be found in gut microbiota bacteria (39, 51–54). 7α-epimerization to UDCA also is produced in gut microbiota bacteria (39).

#### 3.1.3 SCFAs

SCFAs such as acetate, propionate and butyrate are from dietary fiber fermentation in the cecum and colon by gut bacteria (55, 56). Acetate (56–58), butyrate (57, 59, 60) and propionate (57, 59, 61) can be generated by different gut microbiota bacteria.

### 3.2 Education on the immune cells

GMMs such as Trp metabolites, BA metabolites and SCFAs have widely effects on the function and differentiation of immune cells such as CD8 and NK cells, which are involved in NKG2A: HLA-E ICB immunotherapy against tumor. They can affect the function and differentiation of CD8 and NK cells through not only direct, more importantly but also indirect role such as GMMs educated immune cells. These educated immune cells include immune regulatory (suppressive) cells such as rMacs, rDCs, MDSCs, Tregs, Bregs, and also immune inflammatory/effective cells such as TH1, TH2, and TH17, which are related to cytotoxic function of CD8 and NK cells. In addition, the metabolites also have direct effects on the function and differentiation of CD8 and NK cells (**Figure 2**). Notably, the functions of GMMs is highly dependent on concentration and time of exposure (62, 63). However, there is absence of evidence on how quickly the metabolites are metabolized once they are produced by bacteria.

#### 3.2.1 Immunosuppressive cells

GMMs can affect the differentiation and function of immune regulatory (suppressive) cells such as rMacs, Tregs, MDSCs and Bregs, which can negatively regulate the function of CD8 and NK cells in HLA-E: NKG2 blockage immunotherapy against tumors.

##### 3.2.1.1 rMacs

###### 3.2.1.1.1 Trp metabolites

The differentiation and function of rMacs can be regulated by Trp metabolites through their receptor AhR (64). Indeed, the activation of AhR causes reduced inflammatory responses through a Ras-related protein Rac1 (ras-related C3 botulinum toxin substrate 1) ubiquitination-dependent mechanism, which can attenuate AKT (protein kinase B) signaling in endotoxin-activated (LPS) monocyte-derived macrophages (65). Trp metabolites 3-HAA inhibits signaling pathways of LPS mediated PI3K (phosphatidylinositol 3 kinase)/AKT (protein kinase B)/mTOR (mammalian target of rapamycin) and NF-κB (nuclear factor κ gene binding) in LPS-stimulated Raw 264.7 cells and Macs (66). In addition, Trp metabolites also inhibit inflammatory responses also through suppressing histamine production in the Macs (67).

###### 3.2.1.1.2 BA metabolites

BA metabolites have an important role in the differentiation and function of rMacs through their receptors such as TGR5 (GPBAR1), FXR, VDR, LXR, PXR, RORγt and CAR (13, 14). They are essential in maintaining immunosuppressive phenotypes of the Macs via TGR5 (GPBAR1) (68–70). Through activating TGR5 (71) or TGR5-cAMP(adenosine monophosphate)-dependent ubiquitination of NLRP3 (72), secondary BAs DCA and LCA can function as endogenous inhibitors of NLRP3 activation to maintain immunosuppressive phenotypes of macrophages. FXR, another BA receptor also is an important negative regulator of macrophages by directly interacting with NLRP3 and caspase 1 (72). Notably, FXR also activates SHP or SOCS3 (suppressor of cytokine signaling 3), FGF19 (fibroblasts growth factor 19) and CYP450 (Cytochrome P450) to inhibit macrophage inflammation (73–76).

###### 3.2.1.1.3 SCFAs

SCFAs butyrate can inhibit lipopolysaccharide (LPS)-mediated proinflammatory mediators such as IL-6, IL-12 and nitric oxide (NO) in macrophages. The inflammatory responses mediated by NLRP3 are negatively modulated by SCFAs to suppress the Macs (77). Butyrate can also reprogram metabolisms of macrophages toward oxidative phosphorylation, causing an anti-inflammatory phenotype *in vivo* (78).

##### 3.2.1.2 Tregs

###### 3.2.1.2.1 Trp metabolites

The generation and expansion of Tregs can be induced by Trp metabolites mediated regulatory DCs. Trp metabolites such as indole also directly regulate the function of Tregs (79, 80). In addition, Trp metabolite Kyn from gut microbiota can enhance differentiation of Tregs by the activation of AhR (81–84), and increase Foxp3^+^Tregs through direct transactivation and the induction of epigenetic modifications which control Foxp3 transcription (84–86). 3-HAA, a downstream metabolite of Kyn also promotes the generation of Foxp3^+^Treg cells via a nuclear coactivator 7 (NCOA7)-dependent pathway in immunoregulatory dendritic cells (87).

###### 3.2.1.2.2 BA metabolites

The differentiation of Tregs is promoted by secondary BA derivatives isoalloLCA (88–90) via the production of mitochondrial reactive oxygen species (mitoROS), which can increase expression of Foxp3 (88). Nuclear receptor subfamily 4, group A, member 1 (NR4A1) is also necessary for the effect of isoalloLCA on Treg cells *in vitro*-induced T_reg_ (iT_reg_) differentiation (91). IsoalloLCA may result in the increased binding of NR4A1 at the Foxp3 locus, causing Foxp3 gene transcription. In addition, the differentiation of TH17 cells is inhibited by 3-OxoLCA by directly binding to the key transcription factor RORγt (retinoid-related orphan receptor-γt), which finally affects the TH17/Treg balance via administration of 3-oxoLCA to mice (88).

###### 3.2.1.2.3 SCFAs

In Treg cell polarization conditions, SCFAs also promote the conversion of naïve T cells toward Tregs (92). An increased number of extra-thymic Foxp3^+^Tregs could be observed in mice provided with SCFAs (93). In mechanism, SCFA receptor GPR43 on the T cells induces the differentiation of Foxp3^+^ Tregs in histone deacetylase (HDAC)-dependent model (94). The differentiation of Tregs is also promoted through upregulating the histone H3 acetylation of Foxp3 by butyrate in *in vitro* CXCR5^+^Bcl-6^+^Foxp3^+^ T_FR_ (iT_FR_) cell culture system (93). Notably, upon exposure to butyrate, DCs also facilitate the differentiation of Foxp3^+^Tregs, and meanwhile also inhibit the differentiation of naïve T cells into interferon (IFN)γ producing TH1 cells.

##### 3.2.1.3 MDSCs

Notably, intra-tumor increased *F. nucleatum* in patients with colorectal cancer (CRC) is associated with enrichment of MDSCs (95). Gut microbiome can also control accumulation of MDSCs in the context of a benign liver disease or colitis (96). Taurodeoxycholate (TDCA), a BA metabolite also causes increased MDSCs in the spleen of septic mice (97). AhR activation triggers dysbiosis, which in turn regulates induction of MDSCs by injecting 2,3,7,8-tetrachlorodibenzo-p-dioxin directly into antibiotic-treated mice (98).

##### 3.2.1.4 Other immune cells

###### 3.2.1.4.1 Trp metabolites

Trp metabolites also promote differentiation of Tr1 cells, another immune regulatory T cells (99) by activating AhR through associating with c-Maf to activate IL-10 and IL-21 promoters during the differentiation (100). Differentiation and function of IL-10-producing Bregs are also induced by AhR under Breg-inducing conditions (101). IAA together with LPS can activate transcription factor PXR and NF-κB to induce the generation of IL-35^+^ Breg cells *in vitro* culture condition (102). Trp metabolite IAA downregulates RORγt (transcription factors retinoic acid receptor-related orphan receptor gamma t) and STAT3 (signal transducer and activator of transcription 3) to decreases TH17 cells through activating the AhR pathway (103). IAld generated by *Lactobacilli* fosters IL-22 production by ILC3s (104, 105). AhR is an important transcription factor for all ILC3 subsets such as lymphoid tissue-inducer (LTi)-like ILC3s and NKp46^+^ ILC3s (106–108). AhR also promotes ILC3 survival by IL-7/IL-7R pathway and anti-apoptotic gene expression *in vitro* (107), and drives the expression of IL-22 in TH17 cells but is not required for their differentiation in mice (109). In addition, *L. reuteri* can drive reprogramming of CD4^+^ T cells into CD4^+^CD8αα^+^ intestinal intraepithelial lymphocytes (IELs) in the gut via Trp metabolites mediated AhR activation (35). Oral Trp supplementation suppresses antigen-specific TH1 responses at sub-toxic concentrations (110).

###### 3.2.1.4.2 BA metabolites

BA metabolites disrupt intracellular calcium homeostasis, which is essential for NFAT (nuclear factor of activated T cells) signaling in T cells (111). In the presence of BAs, CD4^+^ T effector cells upregulated the xenobiotic transporter Mdr1 in the ileum to maintain homeostasis (112). Pols et al. revealed that physiological concentrations of unconjugated LCA could inhibit the activation of primary human and mouse CD4^+^ TH1 cells through a VDR-dependent mechanism, resulting in decreased TNFα and INFγ (113). PXR activation in both mouse and human also inhibits T cell proliferation *in vitro*. TH17 cell differentiation can be inhibited by 3-oxoLCA by blocking the function of RORγt (88, 114) and directly binding to transcription factor RORγt (88). Similar to 3-oxoLCA, isoLCA also inhibits TH17 cell differentiation by suppressing RORγt (52). The administration of 3-oxoLCA and isoalloLCA to mice can reduce TH17 cell differentiation but increases Treg cell differentiation *in vivo* (88).

###### 3.2.1.4.3 SCFAs

The expression of indoleamine 2,3-dioxygenase 1 (IDO1) and aldehyde dehydrogenase 1A2 (Aldh1A2), immunosuppressive enzymes in DCs, can be induced by butyrate (115). Mouse DCs treated with SCFA propionate exhibit the impaired ability to initiate TH2 effector function (116). In addition, Rosser and colleagues showed that butyrate could cause the production of 5-hydroxyindole-3-acetic acid (5-HIAA) (117), which could activate its AhR in Bregs to mediate the suppressive effect in a rheumatoid arthritis model *in vivo* (117). Administration of SCFAs also increases the frequency of Bregs and improve rheumatoid arthritis (RA) symptoms (118). In addition, SCFA butyrate also decreases the proliferation and cytokine production of TH1, TH17 and TH22 cells (119). Furthermore, SCFAs also deeply impact on ILC function as demonstrated by the effects of antibiotics on the transcriptomic program of ILC1s, ILC2s, and ILC3s (120).

#### 3.2.2 Inflammatory immune cells

GMMs can also affect the differentiation and function of inflammatory/effective immune cells such as inflammatory macrophages (iMAC), TH1 and TH17 cells, which can have positive regulation on the CD8 and NK cells in HLA-E: NKG2 blockage immunotherapy against tumors.

##### 3.2.2.1 Trp metabolites

Trp metabolites promote inflammatory/effective immune cells such as that Trp metabolite ILA ameliorates colorectal tumorigenesis through epigenetic regulation of CD8^+^ T cell immunity (121). Interventions with indole-3-lactic acid (ILA) also complements chemoprevention strategies for colorectal carcinoma (CRCs) (121). Trp metabolite Kyn may modulate GPR35-positive macrophages to cause a robust TH17 immune response.

##### 3.2.2.2 BA metabolites

CDCA, a BA metabolite CDCA suppresses M2 macrophage polarization (122) through causing mitochondrial morphology damage, decreasing mitochondrial membrane potential and elevating mitochondrial calcium level, which can cause the production of ROS.

##### 3.2.2.3 SCFAs

SCFAs increase tumor-killing CD8^+^ T cells and reduce immune-suppressing Tregs in tumor tissues. The supplement using SCFAs increases intra-tumor T cells, raising the concentration of cytokines INF-γ and TNF-α (123). Decreased SCFA-producing taxa such as *Coprococcus* is subsequently related to a lower number of CD8^+^cells. He et al. also exhibited that butyrate could promote draining lymph node CD8^+^ T cells in a mouse model (124). Notably, the evidence of SCFAs for the responses to ICBs is conflicting (62, 124, 125), which needs to be further investigated.

##### 3.2.2.4 Others

Gut microbiota-derived stimulator of interferon gene (STING) agonists such as that cdAMP can induce monocytes to produce type I IFN and to skew the polarization of M1 macrophages in the TME (126). The choline or carnitine in foods are metabolized by the gut microbiota to generate trimethylamine (TMA), which is catalyzed to produce trimethylamine oxide (TMAO). TMAO can promote CD8^+^ T cell-mediated anti-tumor immunity via induction of pyroptosis in mouse models (127). Inosine, a purine metabolite of *A. muciniphila* and *B. pseudolongum*, could act as a substitute carbon source for CD8 cell metabolism in the TME, assisting T-cell proliferation and differentiation to improve sensitivity to ICBs. Other metabolites such as peptidoglycan and polysaccharide, outer membrane vesicles, microbial peptides, anacardic acid and castalagin also promote the differentiation and function of inflammatory cells.

In addition, bacteria such as *Akkermansia muciniphila (A. muciniphila), Bacteroides (B) fragilis*, *Bifidobacterium pseudolongum (B. pseudolongum)*, *Clostridiales* SPP., *Eleven SPP.* and *Lactobacillus* species significantly enhance efficacy of ICBs in the cancer (128–130). They can promote the differentiation and function of inflammatory immune cells. For example, oral administration with *A. muciniphila* recruits CCR9^+^CXCR3^+^CD4^+^ T lymphocyte into tumor (131). *Bifidobacterium* alters the functional capacity of DCs to induce CD8^+^ T cell proliferation (130, 132) and antitumor immune responses to improve ICB efficacy (128)*. Bacteroides fragilis* induces macrophage polarization to M1 and upregulates costimulatory molecule CD80 and CD86 expression on the cells, which can promote innate immunity (133). *L. plantarum* also promotes the expression of natural cytotoxic receptors, and activate NK cells to trigger innate immunity*. Enterococcus hirae* induces the polarization of immune cells towards a TH1 IFNγ phenotype, leading to increased ratios of cytotoxic T cells to Tregs in mouse models. *Faecalibacterium* increases CD4^+^ T cell proportion and also reduces Treg cell proportion in peripheral blood. Thus, gut microbiota/metabolites also educate inflammatory immune cells via promoting their differentiation and function.

## 4 Effects on CD8 and NK cells by GMM educated immune cells

### 4.1 Effects on CD8^+^ T cells by GMM educated immune cells

**Figure 3:**
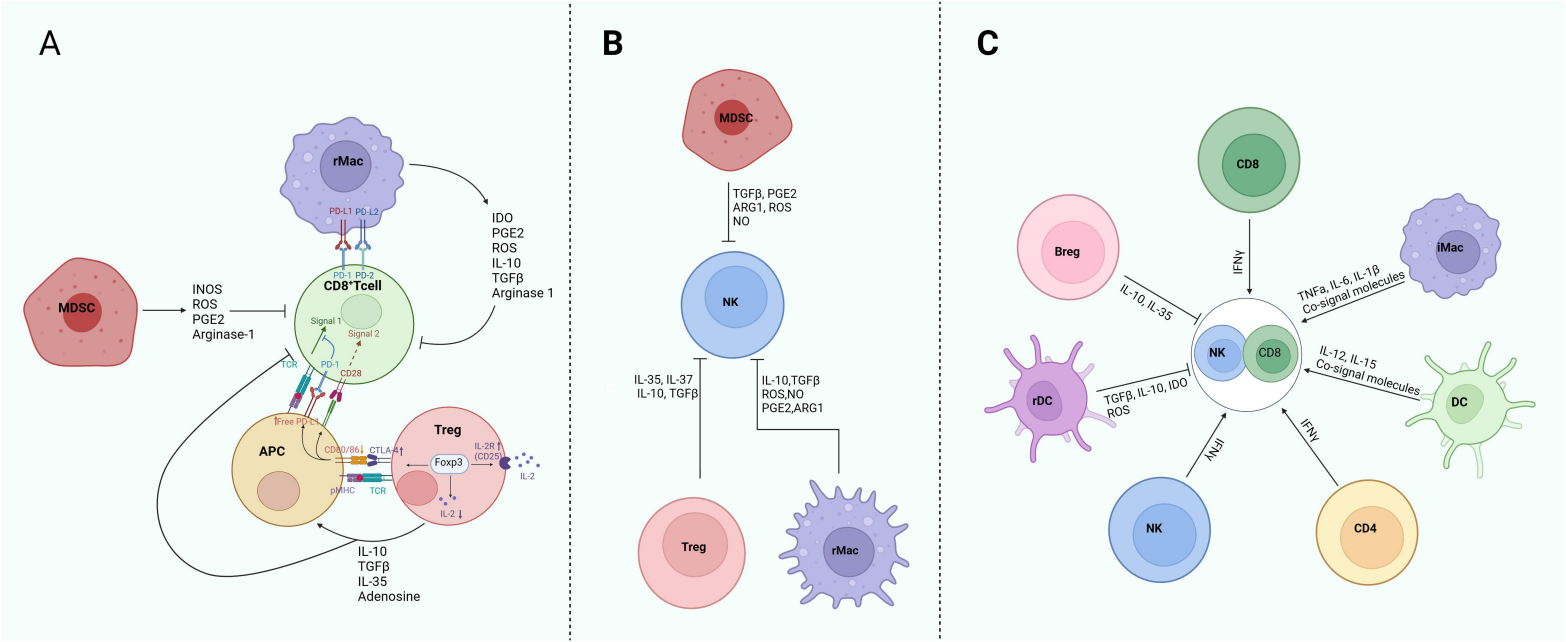
Regulation of GMM educated immune cells on the CD8 and NK cells in HLA-E: NKG2 blockage immunotherapy against tumors. **(A)** Immunosuppression of Tregs, rMacs and MDSCs on the CD8 cells. Down-regulation of CD80 and CD86 co-stimulatory molecules in antigen-presenting cells (APCs) by Tregs to deprive CD8 T cells of CD28 signaling and allowing more free PD-L1 to suppress activated effective T cells through PD-1, which inhibits TCR signaling. Sequestration of IL-2 by Tregs-induced expression of CD25 limits the availability of IL-2 for effective CD8 T cells. Secretion of cytokines, such as IL-10, TGF-β, IL-35 and metabolites adenosine by Tregs directly regulate CD8 T cells and also APC activity. The PD-L1 and PD-L2 expressed on Macs affect CD8 T cell function through PD-1 on the CD8 T cells. MDSCs inhibit CD8 T cells through INOS, ROS, PGE2 and arginase-1. **(B)** Immunosuppression of Tregs, rMacs and MDSCs on the NK cells. Tregs suppress NK cells via IL-35, IL-37, IL10 and TGFβ1; Whereas rMacs inhibit NK cells mainly through cytokines IL-10 and TGFβ, and metabolites NO, ROS, PEG2 and ARG1. MDSCs suppress NK cells through TGFβ1, PGE2, ARG1, ROS and NO. **(C)** Effects of immunosuppressive cells and inflammatory cells on the activity and function of CD8 and NK cells. rMac, regulatory macrophages; Treg, regulatory T cells; Breg, regulatory B cells; rDC, regulatory dendritic cells; MDSC, myeloid-derived suppressive cells; iMac, inflammatory macrophages. PGE2, prostaglandin E2; ARG1, arginase 1; ROS, reactive oxygen species; TGF, tumor growth factor; TNF, tumor necrosis factor; IDO, indoleamine 2,3-dioxygenase; INOS, inducible NO synthase.

GMM educated immunosuppressive immune cells such as Tregs, rMacs and MDSCs can inhibit the activity of effective NK and CD8 cells, but gut microbiota associated inflammatory cells such as iMacs, DCs and TH1 also promote these effective cells through cytokine, metabolites and/or co-signal molecules (27, 134) (**Figure 3**).

Cytotoxic CD8 T cells have positive cross-talking with immune-stimulatory cells such as NK cells, iMacs, CD4^+^ T cells and DCs, but they have also negative cross-talking with immunosuppressive cells such as Tregs, rMacs and MDSCs (27). Tregs-mediated CD8^+^T cell suppression is applied at their both priming and effector phases (135). Major immunosuppressive effects of Tregs on the effective CD8 T cells include cell contact-dependent mechanisms such as down-regulation of CD80 and CD86 to deprive T effective cells of CD28 signal and to allow more free PD-L1 to suppress activated T cells through PD-1, which inhibits TCR signaling. Sequestration of IL-2 by Tregs-induced expression of CD25 can limit the availability of IL-2 for CD8 T cells. The secretion of cytokines such as IL-10, TGF-β, and IL-35, and generation of adenosine also regulate APC activity (136). The cytokines IL-10 and IL-35 by Tregs cooperatively promote intra-tumor T cell exhaustion by modulating multiple inhibitory receptor expression and exhaustion-associated transcriptomic signature of cytotoxic CD8^+^ (137, 138). The release of TGF-β by Tregs can inhibit the expressions for cytolytic gene products from CTLs, including granzymes A and B, perforin, FasL, and IFN-γ (139). In addition, CD73 on the surface of Tregs also contributes to Treg-mediated inhibition of CTL immunosuppressive activity (140). CD73 expression in the T cells is induced by TGF-β (141). Tregs also generate metabolites such as adenosine, prostaglandin E2 (PGE2) and ROS, which are potent suppressors of effector CD8^+^T cells (142). ROS/RNS compounds secreted by Tregs also inhibit the TCR signaling of mouse T cells and cytotoxic activity, cytokine production, and signal transduction of NK cells (143). Indeed, increased frequency of Treg cells and a reduced CD8^+^/Treg cell-ratio in tumors are linked to poor prognosis in multiple cancers (138). Tregs can also kill effector T cells directly in culture through the release of perforins/granzyme B (144).

Gut microbiota mediated Macs such as immunosuppressive Macs or rMacs can regulate activity of effective CD8^+^ T cells. Activated rMacs have anti-inflammatory effects on the CD8^+^ cells (145). These Macs are characterized by a high expression of immune-checkpoint molecules (PDL1, PDL2, B7-H4), which can cause T cell exhaustion. PD-L1 binding to PD-1 in the T cells has been demonstrated to induce apoptosis of T cells, thereby facilitating immune escape (146). Macs with high PD-L1 expression also secreted the typical chemokines, TGF-β and IL-10 (147), which repress the immune system and impair responses from CTLs (148, 149). Secretion of IL-10, TGFβ, prostaglandins and indoleamine 2,3-dioxygenase (IDO) also promotes T cell metabolic starvation. Immunosuppressive Macs or rMacs also produce other immunosuppressive factors such as ROS, prostaglandins, arginase-1 (ARG1), and IDO, which suppress the functions of CD8 T and promote T cell metabolic starvation (150). Elevated expression of the enzyme IDO1 by immunosuppressive Macs results in the consumption of Trp, an amino acid that is essential for the functional activation of T cells.

MDSCs can suppress T cells through ARG1, INOS and ROS (151). Polymorphonuclear neutrophils (PMN) -MDSCs also exerted immunosuppressive effect through S100A8/A9. Mechanically, S100A8/A9 led to CD8^+^ T cells exhaustion including inhibiting CD8^+^ T cells glycolysis, proliferation and TNF-α and IFN-γ production, which was dependent on TLR4/AKT/mTOR pathway (152). MDSCs suppress CD8^+^ T cell activity via the IL-6/IL-8-ARG1 axis in human gastric cancer (153). They promote apoptosis of tumor-infiltrating CD8 T cells and immunotherapy resistance in breast cancer (154).

### 4.2 Effects on NK cells by GMM educated immune cells

Effects of GMMs on NK cells may be realized through GMM-educated immune cells (**Figure 3**). Immunosuppressive Tregs, rMacs and MDSCs actively suppress NK cell-dependent anticancer immunity (155). Both human and mouse canonical NK cells are highly sensitive to Treg cell-mediated immunosuppression, generally resulting in decreased expression of NK cell-activating receptors such as NKG2D, upregulation of co-inhibitory receptors such as PD-1 and interleukin 1 receptor accessory protein like 1 (IL1-RAPL1, best known as IL1R8) to limit proliferative and cytotoxic responses upon activation (156). Tregs also inhibit NK-cell functions either via TGF-β or direct killing (157).

Immunosuppressive Macs inhibit the activation and function of NK cells through cytokines such as IL-10 and TGFβ, and metabolites such as ROS, NO, PEG2 and ARG-1. Notably, monoclonal antibodies targeting scavenger receptors on rMacs can de-repress the cytolytic functions of NK cells in both human and mouse models of melanoma (158).

In mouse tumor model, the frequency of MDSCs inversely correlates with the expression of NK cell-activating receptors including NKG2D and natural cytotoxicity triggering receptor 3 on the NK cell surface, as well as with IFNγ and PRF1 (perforin 1) production (159). At least in preclinical models, the ability of MDSCs to suppress NK cell functions requires physical contact, which is facilitated by membrane-bound TGFβ (160). Additional mechanisms through which MDSCs inhibit NK cells include the production of ROS and reactive nitrogen species, as well as the depletion of essential amino acids such as arginine, reflecting the elevated expression of ARG1 (151, 161). Interactions with regulatory DCs can inhibit NK cells through downregulation of CD80/CD86 on DCs or upregulation of indoleamine 2,3-dioxygenase (IDO) in rDCs (162).

## 5 Direct effects of GMMs on CD8 and NK cells

Notably, gut microbiota/GMMs also exert direct effects on CD8 and NK cells in HLA-E and NKG2 blockage immunotherapy against tumors though inhibiting or promoting their differentiation and function. For example, *F. nucleatum* can inhibit the attack of natural killer (NK) cells on tumor cells by binding TIGIT (T cell immunoglobulin and ITIM domain), an inhibitory receptor on human NK cells and various T cells via the fusobacterial Fap2 protein (163). Gut microbes promotes pancreatic ductal adenocarcinoma by decreasing the intratumoral infiltration and activity of NK cells (164). *Eleven* strain combined with ICBs also induces IFN γ^+^CD8^+^T cells to inhibit tumor growth. Hezaveh et al. showed that indole compounds, tryptophan metabolites by *Lactobacillus*, could activate the aryl hydrocarbon receptor in tumor-associated macrophages to inhibits intratumoral infiltration of CD8 ^+^ T cells in the pancreatic ductal adenocarcinoma (165). SCFAs may limit the antitumor effects of CTLA-4 blockade. High concentration of butyrate in cancer patients can decrease the anticancer activity of ipilimumab by inhibiting the accumulation of T cells (62). Notably, PD-1 expression in effective CD8^+^T cells is also upregulated through ligand-activated AhR (166). 5-hydroxytryptophan (5-HTP) subsequently activates AhR nuclear translocation, causing a coordinated upregulation of inhibitory receptors and downregulation of cytokine and effector-molecule production, thereby rendering T cells dysfunctional in the tumor microenvironment (167). The immune suppression can also be caused by 3-HAA by inducing apoptosis in T-cells (168). L-kyn metabolites (169) may cause cell death of NK cells via ROS pathway (170). Taken together, the function of immune effective cell CD8 may be directly inhibited by gut microbiota/metabolites.

On the other hand, the activity of CD8 and NK cells can also be promoted by gut microbiota/metabolites. *Bifidobacterium* plays antitumor roles by inducing the maturation of dendritic cells, stimulating cytotoxic CD8 ^+^ T cells (132). Kassayová et al. found that *Lactobacillus (L). plantarum* inhibited the proliferation of breast cancer cells by increasing the levels of CD8^+^ T cells and CD4^+^ T cells (171). *Akkermansia muciniphila* raises the CD8^+^ T proportion to promote liver tumor cells apoptosis in the tumor microenvironment (172). Trp metabolite indole-3-aldehyde (I3A) locally promotes interferon-γ-producing CD8 T cells, thereby bolstering ICB (173) to improve ICB efficacy and survival in advanced melanoma patients. Notably, one study showed that high tryptophan metabolism group had an increased proportion of CD8^+^ T cells, augmented cytolytic activity mediated by CD8^+^ T cells, and promoted expression of immune checkpoint molecules (174). The restoration of gut microbial butyrate also enhance CD8^+^ T cell cytotoxicity via GPR109A/HOPX, inhibiting gastric carcinogenesis (GC) (175).

## 6 Regulation of GMM associated immune factors in NKG2A/CD94 and HLA-E expression

**Figure 4:**
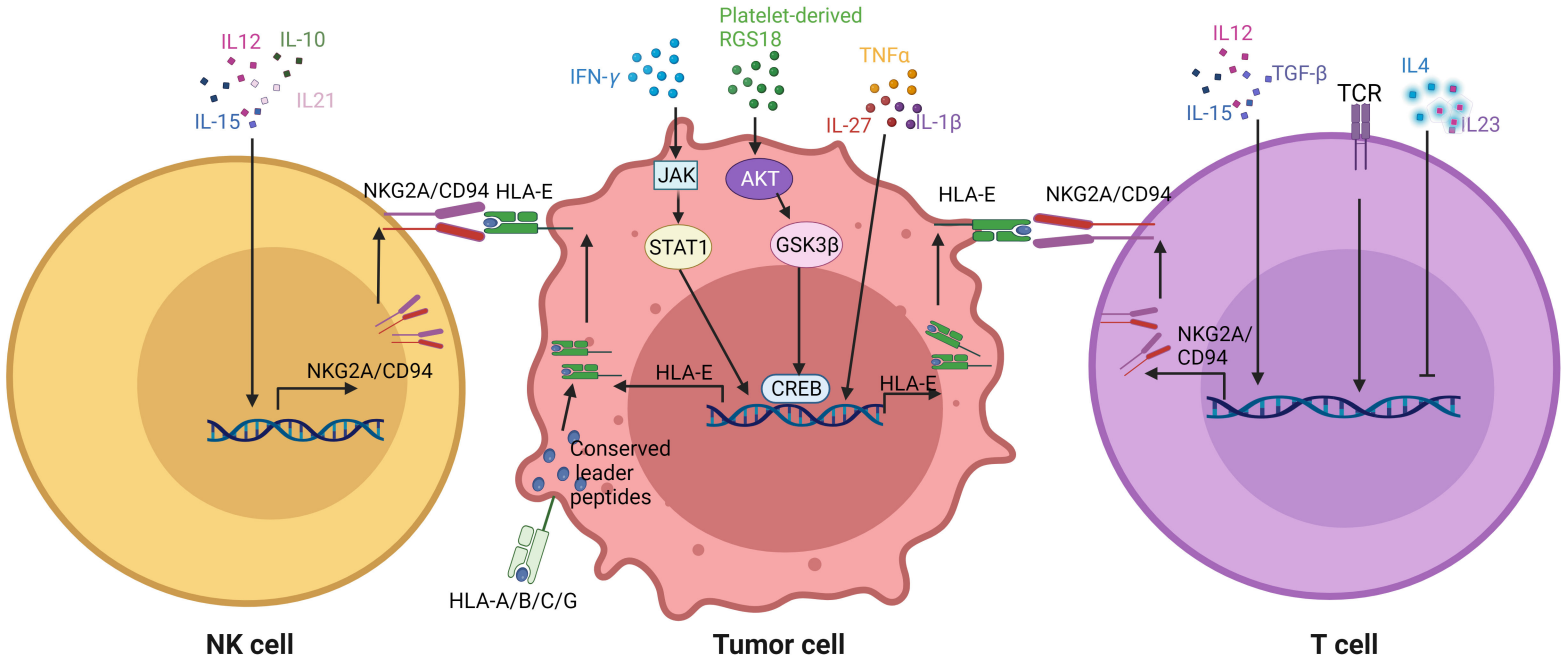
Effects of GMM associated immune factors on the expression of NKG2A/CD94 in immune cells and HLA-E in tumor cells. Immune factors such as cytokines affect the expression of NKG2A/CD94 in the CD8 and NK cells, and also expression of HLA-E in the tumor cells. TCR, T cell receptors; RGS, regulator of G protein signaling; JAK, Janus kinase; STAT1, signal transducers and activators of transcription 1; AKT, protein kinase B; GSK3β, glycogen synthase kinase-3; CREB, cAMP-response element binding protein.

Tumor cells such as HLA-E and immune cells such as CD8 and NK cells can express immune checkpoint molecules upon exposure to immune factors such as cytokines, which prevent NK and CD8 cell activation, even resulting in NK and CD8 cell dysfunction or exhaustion (**Figure 4**).

### 6.1 HLA-E expression in tumor cells

HLA-E expression in tumor cells can be regulated in response to immune associated factors such as IFNγ, TNFα, IL-1β and IL-27, which are also associated to GMM educated immune cells. The IFNγ/JAK/STAT1 pathway plays a crucial role in the antigen processing pathway and the subsequent dynamic change of downstream signals, including major histocompatibility complex (MHC) class I (176). IFNγ, as an important cytokine, promotes HLA-E expression through binding of a STAT1-containing complex to IFNγ-responsive region of the *HLA-E* gene. Higher STAT1 expression is an indicative of high expression levels of MHC class I and PD-L1 in human colon cancer cells *in vitro* (177). Other pro-inflammatory cytokines such as IL-1β, TNF*α* and IL-27 also induce HLA-E expression *in vitro* (178). Notably, recent findings indicate that the platelet-derived RGS18 promotes the expression of HLA-E through AKT-GSK3β-CREB signaling (26). The overexpression of RGS18 facilitates pancreatic tumor hepatic metastasis (26). HLA-E expression is also induced by senescence- related pro-inflammatory cytokines through p38 MAP kinase signaling *in vitro* (179). Notably, HLA-E surface expression is post-translationally regulated by the conserved leader peptides, the peptide transporter TAP, and proteolytic enzymes (180). Their signal sequences contain a highly conserved segment that is eventually presented at the cell surface by the nonpolymorphic nonclassical MHC class I molecule HLA-E. Expression of *HLA-A*, *B* and *C* alleles on tumors promotes higher HLA-E cell-surface expression through provision of VL9 peptides. Furthermore, HLA class I signal peptide polymorphism determines the level of NKG2/CD94-HLA-E-mediated regulation of effector cell responses (181). In mice, the inhibitory CD94/NKG2A receptor recognizes H2-T23 (Qa-1) complexes with leader peptides from H-2D alleles. However, HLA-E expression in tumor microenvironment (TME) does not also always depend on the expression of *HLA-A*, *B* and *C* alleles. Notably, immune cells such as Macs and DCs can contribute to HLA-E enrichment in the TME (11, 15). Thus it is necessary to further investigate the mechanism of HLA-E expression in the TME, which is independent on the HLA-A, B and C alleles.

### 6.2 CD94/NKG2A expression in immune cells

NKG2A expression can be detected in cytotoxic lymphocytes, including most NK cells and a subset of CD8^+^ T cells (182). Multiple cytokines including interleukin (IL)-21, IL-15, IL-12, IL-10 and transforming growth factor β (TGFβ) are able to induce the expression of NKG2A in NK cells (183). IL-12, secreted by Macs and dendritic cells, is an important activator of immune responses against tumor cells, including the generation of NK cells with memory-like properties. TGFβ is often overtly present in the tumor microenvironment (184). IL-15-induced maturation of human NK cells from early thymic precursors can selectively cause the expression of CD94/NKG2A (185).

NKG2A expression in CD8^+^ T cells is highly regulated, differing from its expression pattern in NK cells. A number of cytokines such as IL-12, IL-15, IL-10, IL-6, IL-2 and TGFβ can regulate the expression of NKG2A in CD8^+^ T cells. NKG2A expression may be induced in T cells by TCR triggering in combination with tissue-released cytokines (186, 187), such as the presence of cytokines like IL-15 and TGFβ (188, 189). NKG2A is also expressed by T lymphocytes, either upon prolonged stimulation via TCR (190) or upon exposure to TGF-β, an immunosuppressive cytokine. Indeed, high expression of NKG2A on NK cells and cytotoxic CD8 T cells happens in the tumor microenvironment as a result of PD-1 blockade therapy as well as after immune activation by cancer vaccines (7). However, CD94/NKG2A expression is impaired by several other cytokines such as IL-4 and IL-23. T cells responding to toxic shock syndrome toxin 1 also promote the expression of CD94 in the presence of IL-15 (190). Thus, immune cytokines exert an important role in the expression of CD94/NKG2A of NK and CD8 T cells.

## 7 Enhancing HLA-E: NKG2a blockage immunotherapy against tumors by gut microbiota

Immune checkpoint inhibitors such as HLA-E: NKG2a blockages are effective immunotherapy strategies against tumor (191). However, the interactions between gut microbiota and immune cells can affect tumor immunotherapy. In melanoma patients, the diversity and composition of gut microbiota were positively correlated with anti-PD-1 therapy responses (130). *B. longum*, *Collinsella aerofaciens*, and *Enterococcus faecium* were more abundant in the baseline feces of responders to immune checkpoint inhibitor (192). This causes the development of multiple therapeutic methods targeting on microbiota such as fecal microbiota transplantation (FMT), probiotics, prebiotics, genetically engineered probiotics and phage-targeted depletion of pathogenic bacteria (193, 194). FMT can alter the gut microbiome of patients with cancer to improve the efficacy of immunotherapy such as anti-PD-1 monoclonal antibody (195, 196). Probiotics and/or bacteria consortia containing live bacteria such as *Bifidobacteria, La*c*tobacillus*, *Propionibacterium* and *Streptococcus thermophiles* combined with monoclonal antibodies (anti-PD-1 and anti-CTLA-4 antibodies) also significantly improve the outcomes of cancer patients with immunotherapy. Prebiotics such as oligofructose and inulin are capable of facilitating the growth of healthier microorganisms. Their metabolites such as SCFAs, which enhance tumor cell killing efficacy through promoting effective T lymphocytes. The specific gut bacteria, such as *Bifidobacterium*, *Akkermansia*, *Enterococcus*, *Faecalibacterium*, and *Ruminococcaceae*, play the role of immune adjuvants in ICB immunotherapy based on anti-PD-1 and anti-CTLA-4 antibodies (197). Although there also are not reports on the involvement of gut microbiota in HLA-E: NKG2A ICB against tumors, it is possible to improve HLA-E: NKG2A ICB immunotherapy against tumors through regulating the composition of gut microbiota. Notably, studies found the bacteria universally linked to ICB resistance (198). Future studies will help sharpen the effect of the specific bacteria and their potential as new biomarkers.

## 8 Conclusion and perspectives

Blocking the interaction of NKG2A expressed on both NK cells and CD8^+^ T cells with HLA-E in tumor cells is effective in immunotherapy against tumor. The activity of the NK cells and CD8^+^ T cells can be regulated not only by GMM educated immune cells but also directly by GMM. Furthermore, the expression of HLA-E in tumor cells and CD94/NKG2A in CD8 and NK cells are also affected by GMM associated immune factors. These findings offer new insights for the clinical application of gut microbiota/metabolites in precise and/or personalized treatments of tumors. Indeed, immunotherapy against tumor based HLA-E: NKG2A might be improved through diet, fecal bacterial transplantation, probiotics (individual probiotics or cocktails), prebiotics (dietary fiber and the related metabolites), genetically engineered probiotics and phage-targeted depletion of pathogenic bacteria (193, 194). Notably, the levels of bacteria in tumor tissues might not have anything to do with their abundance in the gut. Although there have many challenges, the full potential of gut microbiota/metabolites cannot be overstated for the new anti-tumor strategies. But, it is critical to decipher the specialized roles of GMMs in regulating the immune cells in tumors.

## References

[1] Sharma P Goswami S Raychaudhuri D Siddiqui BA Singh P Nagarajan A. Immune checkpoint therapy-current perspectives and future directions. Cell. (2023) 186:1652–69. doi: 10.1016/j.cell.2023.03.006 37059068

[2] Kanannejad Z Soleimanian S Ghahramani Z Sepahi N Mohkam M Alyasin S. Immune checkpoint molecules in prevention and development of asthma. Front Immunol. (2023) 14:1070779. doi: 10.3389/fimmu.2023.1070779 36865540 PMC9972681

[3] Talebian Yazdi M van Riet S van SChadewijk A Fiocco M van Hall T Taube C. The positive prognostic effect of stromal CD8+ tumor-infiltrating T cells is restrained by the expression of HLA-E in non-small cell lung carcinoma. Oncotarget. (2016) 7:3477–88. doi: 10.18632/oncotarget.6506 PMC4823121 26658106

[4] Seliger B Jasinski-Bergner S Quandt D Stoehr C Bukur J Wach S. HLA-E expression and its clinical relevance in human renal cell carcinoma. Oncotarget. (2016) 7:67360–72. doi: 10.18632/oncotarget.11744 PMC5341881 27589686

[5] Andersson E Poschke I Villabona L Carlson JW Lundqvist A Kiessling R. Non-classical HLA-class I expression in serous ovarian carcinoma: Correlation with the HLA-genotype, tumor infiltrating immune cells and prognosis. Oncoimmunology. (2016) 5:e1052213. doi: 10.1080/2162402X.2015.1052213 26942060 PMC4760332

[6] Borst L van der Burg SH van Hall T. The NKG2A-HLA-E axis as a novel checkpoint in the tumor microenvironment. Clin Cancer Res. (2020) 26:5549–56. doi: 10.1158/1078-0432.CCR-19-2095 32409305

[7] Andre P Denis C Soulas C Bourbon-Caillet C Lopez J Arnoux T. Anti-NKG2A mAb is a checkpoint inhibitor that promotes anti-tumor immunity by unleashing both T and NK cells. Cell. (2018) 175:1731–43 e13. doi: 10.1016/j.cell.2018.10.014 30503213 PMC6292840

[8] Salome B Sfakianos JP Ranti D Daza J Bieber C Charap A. NKG2A and HLA-E define an alternative immune checkpoint axis in bladder cancer. Cancer Cell. (2022) 40:1027–43 e9. doi: 10.1016/j.ccell.2022.08.005 36099881 PMC9479122

[9] Liu W Sheng S Zhu C Li C Zou Y Yang C. Increased NKG2A(+)CD8(+) T-cell exhaustion in patients with adenomyosis. Mucosal Immunol. (2023) 16:121–34. doi: 10.1016/j.mucimm.2023.02.003 36828189

[10] Battaglia NG Murphy JD Uccello TP Hughson A Gavras NW Caldon JJ. Combination of NKG2A and PD-1 blockade improves radiotherapy response in radioresistant tumors. J Immunol. (2022) 209:629–40. doi: 10.4049/jimmunol.2100044 PMC9339479 35840162

[11] Abd Hamid M Wang RZ Yao X Fan P Li X Chang XM. Enriched HLA-E and CD94/NKG2A interaction limits antitumor CD8(+) tumor-infiltrating T lymphocyte responses. Cancer Immunol Res. (2019) 7:1293–306. doi: 10.1158/2326-6066.CIR-18-0885 31213473

[12] Gimeno L Gonzalez-Lozano I Soto-Ramirez MF Martinez-Sanchez MV Lopez-Cubillana P Fuster JL. CD8+ T lymphocytes are sensitive to NKG2A/HLA-E licensing interaction: role in the survival of cancer patients. Oncoimmunology. (2021) 10:1986943. doi: 10.1080/2162402X.2021.1986943 34676148 PMC8525952

[13] Wang J Zhu N Su X Gao Y Yang R. Gut-microbiota-derived metabolites maintain gut and systemic immune homeostasis. Cells. (2023) 12:793. doi: 10.3390/cells12050793 36899929 PMC10000530

[14] Su X Gao Y Yang R. Gut microbiota-derived tryptophan metabolites maintain gut and systemic homeostasis. Cells. (2022) 11:2296. doi: 10.3390/cells11152296 35892593 PMC9330295

[15] Jiang H Yu D Yang P Guo R Kong M Gao Y. Revealing the transcriptional heterogeneity of organ-specific metastasis in human gastric cancer using single-cell RNA Sequencing. Clin Transl Med. (2022) 12:e730. doi: 10.1002/ctm2.730 35184420 PMC8858624

[16] Kaiser BK Pizarro JC Kerns J Strong RK. Structural basis for NKG2A/CD94 recognition of HLA-E. Proc Natl Acad Sci U S A. (2008) 105:6696–701. doi: 10.1073/pnas.0802736105 PMC2373352 18448674

[17] Borst L Sluijter M Sturm G Charoentong P Santegoets SJ van Gulijk M. NKG2A is a late immune checkpoint on CD8 T cells and marks repeated stimulation and cell division. Int J Cancer. (2022) 150:688–704. doi: 10.1002/ijc.33859 34716584 PMC9299709

[18] Fang Y Bian C Li Z Jin L Chen C Miao Y. ScRNA-seq revealed disruption in CD8(+) NKG2A(+) natural killer T cells in patients after liver transplantation and immunosuppressive therapy. Immun Inflammation Dis. (2023) 11:e990. doi: 10.1002/iid3.990 PMC10524014 37773707

[19] Viant C Fenis A Chicanne G Payrastre B Ugolini S Vivier E. SHP-1-mediated inhibitory signals promote responsiveness and anti-tumour functions of natural killer cells. Nat Commun. (2014) 5:5108. doi: 10.1038/ncomms6108 25355530

[20] Long EO Kim HS Liu D Peterson ME Rajagopalan S. Controlling natural killer cell responses: integration of signals for activation and inhibition. Annu Rev Immunol. (2013) 31:227–58. doi: 10.1146/annurev-immunol-020711-075005 PMC3868343 23516982

[21] van Montfoort N Borst L Korrer MJ Sluijter M Marijt KA Santegoets SJ. NKG2A blockade potentiates CD8 T cell immunity induced by cancer vaccines. Cell. (2018) 175:1744–55 e15. doi: 10.1016/j.cell.2018.10.028 30503208 PMC6354585

[22] Chen Y Xin Z Huang L Zhao L Wang S Cheng J. CD8(+) T cells form the predominant subset of NKG2A(+) cells in human lung cancer. Front Immunol. (2019) 10:3002. doi: 10.3389/fimmu.2019.03002 32010126 PMC6979261

[23] Kamiya T Seow SV Wong D Robinson M Campana D. Blocking expression of inhibitory receptor NKG2A overcomes tumor resistance to NK cells. J Clin Invest. (2019) 129:2094–106. doi: 10.1172/JCI123955 PMC6486333 30860984

[24] Wang X Cui Y Luo G Wang Q Hu J He W. Activated mouse CD4(+)Foxp3(-) T cells facilitate melanoma metastasis *via* Qa-1-dependent suppression of NK-cell cytotoxicity. Cell Res. (2012) 22:1696–706. doi: 10.1038/cr.2012.128 PMC3515753 22945357

[25] Zhang C Wang XM Li SR Twelkmeyer T Wang WH Zhang SY. NKG2A is a NK cell exhaustion checkpoint for HCV persistence. Nat Commun. (2019) 10:1507. doi: 10.1038/s41467-019-09212-y 30944315 PMC6447531

[26] Liu X Song J Zhang H Liu X Zuo F Zhao Y. Immune checkpoint HLA-E:CD94-NKG2A mediates evasion of circulating tumor cells from NK cell surveillance. Cancer Cell. (2023) 41:272–87 e9. doi: 10.1016/j.ccell.2023.01.001 36706761

[27] Farhood B Najafi M Mortezaee K. CD8(+) cytotoxic T lymphocytes in cancer immunotherapy: A review. J Cell Physiol. (2019) 234:8509–21. doi: 10.1002/jcp.27782 30520029

[28] Agus A Planchais J Sokol H. Gut microbiota regulation of tryptophan metabolism in health and disease. Cell Host Microbe. (2018) 23:716–24. doi: 10.1016/j.chom.2018.05.003 29902437

[29] Roager HM Licht TR. Microbial tryptophan catabolites in health and disease. Nat Commun. (2018) 9:3294. doi: 10.1038/s41467-018-05470-4 30120222 PMC6098093

[30] Gao J Xu K Liu H Liu G Bai M Peng C. Impact of the gut microbiota on intestinal immunity mediated by tryptophan metabolism. Front Cell Infect Microbiol. (2018) 8:13. doi: 10.3389/fcimb.2018.00013 29468141 PMC5808205

[31] Dodd D Spitzer MH Van Treuren W Merrill BD Hryckowian AJ Higginbottom SK. A gut bacterial pathway metabolizes aromatic amino acids into nine circulating metabolites. Nature. (2017) 551:648–52. doi: 10.1038/nature24661 PMC5850949 29168502

[32] Lamas B Richard ML Leducq V Pham HP Michel ML Da Costa G. CARD9 impacts colitis by altering gut microbiota metabolism of tryptophan into aryl hydrocarbon receptor ligands. Nat Med. (2016) 22:598–605. doi: 10.1038/nm.4102 27158904 PMC5087285

[33] Russell WR Duncan SH Scobbie L Duncan G Cantlay L Calder AG. Major phenylpropanoid-derived metabolites in the human gut can arise from microbial fermentation of protein. Mol Nutr Food Res. (2013) 57:523–35. doi: 10.1002/mnfr.201200594 23349065

[34] Wlodarska M Luo C Kolde R d'Hennezel E Annand JW Heim CE. Indoleacrylic acid produced by commensal peptostreptococcus species suppresses inflammation. Cell Host Microbe. (2017) 22:25–37 e6. doi: 10.1016/j.chom.2017.06.007 28704649 PMC5672633

[35] Cervantes-Barragan L Chai JN Tianero MD Di Luccia B Ahern PP Merriman J. Lactobacillus reuteri induces gut intraepithelial CD4(+)CD8alphaalpha(+) T cells. Science. (2017) 357:806–10. doi: 10.1126/science.aah5825 PMC5687812 28775213

[36] Smith EA Macfarlane GT. Enumeration of human colonic bacteria producing phenolic and indolic compounds: effects of pH, carbohydrate availability and retention time on dissimilatory aromatic amino acid metabolism. J Appl Bacteriol. (1996) 81:288–302. doi: 10.1111/j.1365-2672.1996.tb04331.x 8810056

[37] Vujkovic-Cvijin I Dunham RM Iwai S Maher MC Albright RG Broadhurst MJ. Dysbiosis of the gut microbiota is associated with HIV disease progression and tryptophan catabolism. Sci Transl Med. (2013) 5:193ra91. doi: 10.1126/scitranslmed.3006438 PMC4094294 23843452

[38] Lucas LN Barrett K Kerby RL Zhang Q Cattaneo LE Stevenson D. Dominant bacterial phyla from the human gut show widespread ability to transform and conjugate bile acids. mSystems. (2021):e0080521. doi: 10.1128/mSystems.00805-21 34463573 PMC12338150

[39] Guzior DV Quinn RA. Review: microbial transformations of human bile acids. Microbiome. (2021) 9:140. doi: 10.1186/s40168-021-01101-1 34127070 PMC8204491

[40] Harris SC Devendran S Mendez-Garcia C Mythen SM Wright CL Fields CJ. Bile acid oxidation by Eggerthella lenta strains C592 and DSM 2243(T). Gut Microbes. (2018) 9:523–39. doi: 10.1080/19490976.2018.1458180 PMC6287680 29617190

[41] Eggert T Bakonyi D Hummel W. Enzymatic routes for the synthesis of ursodeoxycholic acid. J Biotechnol. (2014) 191:11–21. doi: 10.1016/j.jbiotec.2014.08.006 25131646

[42] Bourgin M Kriaa A Mkaouar H Mariaule V Jablaoui A Maguin E. Bile salt hydrolases: at the crossroads of microbiota and human health. Microorganisms. (2021) 9:1122. doi: 10.3390/microorganisms9061122 34067328 PMC8224655

[43] Foley MH O'Flaherty S Allen G Rivera AJ Stewart AK Barrangou R. Lactobacillus bile salt hydrolase substrate specificity governs bacterial fitness and host colonization. Proc Natl Acad Sci U S A. (2021) 118:e2017709118. doi: 10.1073/pnas.2017709118 33526676 PMC8017965

[44] Coleman JP Hudson LL. Cloning and characterization of a conjugated bile acid hydrolase gene from Clostridium perfringens. Appl Environ Microbiol. (1995) 61:2514–20. doi: 10.1128/aem.61.7.2514-2520.1995 PMC167523 7618863

[45] Gopal-Srivastava R Hylemon PB. Purification and characterization of bile salt hydrolase from Clostridium perfringens. J Lipid Res. (1988) 29:1079–85. 2903208

[46] Xiao Y Zhao J Zhang H Zhai Q Chen W. Mining genome traits that determine the different gut colonization potential of Lactobacillus and Bifidobacterium species. Microb Genom. (2021) 7:000581. doi: 10.1099/mgen.0.000581 34100697 PMC8461469

[47] Bhagwat A Annapure US. *In vitro* assessment of metabolic profile of Enterococcus strains of human origin. J Genet Eng Biotechnol. (2019) 17:11. doi: 10.1186/s43141-019-0009-0 31761970 PMC6875533

[48] Adhikari AA Seegar TCM Ficarro SB McCurry MD Ramachandran D Yao L. Development of a covalent inhibitor of gut bacterial bile salt hydrolases. Nat Chem Biol. (2020) 16:318–26. doi: 10.1038/s41589-020-0467-3 PMC7036035 32042200

[49] Devlin AS Fischbach MA. A biosynthetic pathway for a prominent class of microbiota-derived bile acids. Nat Chem Biol. (2015) 11:685–90. doi: 10.1038/nchembio.1864 PMC4543561 26192599

[50] Fiorucci S Carino A Baldoni M Santucci L Costanzi E Graziosi L. Bile acid signaling in inflammatory bowel diseases. Dig Dis Sci. (2021) 66:674–93. doi: 10.1007/s10620-020-06715-3 PMC7935738 33289902

[51] Sato Y Atarashi K Plichta DR Arai Y Sasajima S Kearney SM. Novel bile acid biosynthetic pathways are enriched in the microbiome of centenarians. Nature. (2021) 599:458–64. doi: 10.1038/s41586-021-03832-5 34325466

[52] Paik D Yao L Zhang Y Bae S D'Agostino GD Zhang M. Human gut bacteria produce TauEta17-modulating bile acid metabolites. Nature. (2022) 603:907–12. doi: 10.1038/s41586-022-04480-z PMC9132548 35296854

[53] Lee JY Arai H Nakamura Y Fukiya S Wada M Yokota A. Contribution of the 7beta-hydroxysteroid dehydrogenase from Ruminococcus gnavus N53 to ursodeoxycholic acid formation in the human colon. J Lipid Res. (2013) 54:3062–9. doi: 10.1194/jlr.M039834 PMC3793610 23729502

[54] Ferrandi EE Bertolesi GM Polentini F Negri A Riva S Monti D. In search of sustainable chemical processes: cloning, recombinant expression, and functional characterization of the 7alpha- and 7beta-hydroxysteroid dehydrogenases from Clostridium absonum. Appl Microbiol Biotechnol. (2012) 95:1221–33. doi: 10.1007/s00253-011-3798-x 22198717

[55] Ratajczak W Ryl A Mizerski A Walczakiewicz K Sipak O Laszczynska M. Immunomodulatory potential of gut microbiome-derived short-chain fatty acids (SCFAs). Acta Biochim Pol. (2019) 66:1–12. doi: 10.18388/abp.2018_2648 30831575

[56] Koh A De Vadder F Kovatcheva-Datchary P Backhed F. From dietary fiber to host physiology: short-chain fatty acids as key bacterial metabolites. Cell. (2016) 165:1332–45. doi: 10.1016/j.cell.2016.05.041 27259147

[57] Louis P Hold GL Flint HJ. The gut microbiota, bacterial metabolites and colorectal cancer. Nat Rev Microbiol. (2014) 12:661–72. doi: 10.1038/nrmicro3344 25198138

[58] Rey FE Faith JJ Bain J Muehlbauer MJ Stevens RD Newgard CB. Dissecting the in *vivo* metabolic potential of two human gut acetogens. J Biol Chem. (2010) 285:22082–90. doi: 10.1074/jbc.M110.117713 PMC2903421 20444704

[59] Reichardt N Duncan SH Young P Belenguer A McWilliam Leitch C Scott KP. Phylogenetic distribution of three pathways for propionate production within the human gut microbiota. ISME J. (2014) 8:1323–35. doi: 10.1038/ismej.2014.14 PMC4030238 24553467

[60] Vital M Howe AC Tiedje JM. Revealing the bacterial butyrate synthesis pathways by analyzing (meta)genomic data. mBio. (2014) 5:e00889. doi: 10.1128/mBio.00889-14 24757212 PMC3994512

[61] Scott KP Martin JC Campbell G Mayer CD Flint HJ. Whole-genome transcription profiling reveals genes up-regulated by growth on fucose in the human gut bacterium "Roseburia inulinivorans. J Bacteriol. (2006) 188:4340–9. doi: 10.1128/JB.00137-06 PMC1482943 16740940

[62] Coutzac C Jouniaux JM Paci A Schmidt J Mallardo D Seck A. Systemic short chain fatty acids limit antitumor effect of CTLA-4 blockade in hosts with cancer. Nat Commun. (2020) 11:2168. doi: 10.1038/s41467-020-16079-x 32358520 PMC7195489

[63] Cummings JH Pomare EW Branch WJ Naylor CP Macfarlane GT. Short chain fatty acids in human large intestine, portal, hepatic and venous blood. Gut. (1987) 28:1221–7. doi: 10.1136/gut.28.10.1221 PMC1433442 3678950

[64] Goudot C Coillard A Villani AC Gueguen P Cros A Sarkizova S. Aryl Hydrocarbon Receptor Controls Monocyte Differentiation into Dendritic Cells versus Macrophages. Immunity. (2017) 47:582–96 e6. doi: 10.1016/j.immuni.2017.08.016 28930664

[65] Grosskopf H Walter K Karkossa I von Bergen M Schubert K. Non-genomic ahR-signaling modulates the immune response in endotoxin-activated macrophages after activation by the environmental stressor baP. Front Immunol. (2021) 12:620270. doi: 10.3389/fimmu.2021.620270 33868237 PMC8045971

[66] Lee K Kwak JH Pyo S. Inhibition of LPS-induced inflammatory mediators by 3-hydroxyanthranilic acid in macrophages through suppression of PI3K/NF-kappaB signaling pathways. Food Funct. (2016) 7:3073–82. doi: 10.1039/c6fo00187d 27264984

[67] Masuda K Kimura A Hanieh H Nguyen NT Nakahama T Chinen I. Aryl hydrocarbon receptor negatively regulates LPS-induced IL-6 production through suppression of histamine production in macrophages. Int Immunol. (2011) 23:637–45. doi: 10.1093/intimm/dxr072 21930594

[68] Fiorucci S Biagioli M Zampella A Distrutti E. Bile acids activated receptors regulate innate immunity. Front Immunol. (2018) 9:1853. doi: 10.3389/fimmu.2018.01853 30150987 PMC6099188

[69] Kawamata Y Fujii R Hosoya M Harada M Yoshida H Miwa M. A G protein-coupled receptor responsive to bile acids. J Biol Chem. (2003) 278:9435–40. doi: 10.1074/jbc.M209706200 12524422

[70] Haselow K Bode JG Wammers M Ehlting C Keitel V Kleinebrecht L. Bile acids PKA-dependently induce a switch of the IL-10/IL-12 ratio and reduce proinflammatory capability of human macrophages. J Leukoc Biol. (2013) 94:1253–64. doi: 10.1189/jlb.0812396 23990628

[71] Shi Y Su W Zhang L Shi C Zhou J Wang P. TGR5 regulates macrophage inflammation in nonalcoholic steatohepatitis by modulating NLRP3 inflammasome activation. Front Immunol. (2020) 11:609060. doi: 10.3389/fimmu.2020.609060 33692776 PMC7937818

[72] Hao H Cao L Jiang C Che Y Zhang S Takahashi S. Farnesoid X receptor regulation of the NLRP3 inflammasome underlies cholestasis-associated sepsis. Cell Metab. (2017) 25:856–67.e5. doi: 10.1016/j.cmet.2017.03.007 28380377 PMC6624427

[73] Bertolini A Fiorotto R Strazzabosco M. Bile acids and their receptors: modulators and therapeutic targets in liver inflammation. Semin Immunopathol. (2022) 44:547–64. doi: 10.1007/s00281-022-00935-7 PMC9256560 35415765

[74] Chanda D Park JH Choi HS. Molecular basis of endocrine regulation by orphan nuclear receptor Small Heterodimer Partner. Endocr J. (2008) 55:253–68. doi: 10.1507/endocrj.k07e-103 17984569

[75] Fiorucci S Distrutti E. Bile acid-activated receptors, intestinal microbiota, and the treatment of metabolic disorders. Trends Mol Med. (2015) 21:702–14. doi: 10.1016/j.molmed.2015.09.001 26481828

[76] Yang CS Kim JJ Kim TS Lee PY Kim SY Lee HM. Small heterodimer partner interacts with NLRP3 and negatively regulates activation of the NLRP3 inflammasome. Nat Commun. (2015) 6:6115. doi: 10.1038/ncomms7115 25655831 PMC4347017

[77] Shao X Sun S Zhou Y Wang H Yu Y Hu T. Bacteroides fragilis restricts colitis-associated cancer *via* negative regulation of the NLRP3 axis. Cancer Lett. (2021) 523:170–81. doi: 10.1016/j.canlet.2021.10.002 34627951

[78] Scott NA Andrusaite A Andersen P Lawson M Alcon-Giner C Leclaire C. Antibiotics induce sustained dysregulation of intestinal T cell immunity by perturbing macrophage homeostasis. Sci Transl Med. (2018) 10::eaao4755. doi: 10.1126/scitranslmed.aao4755 30355800 PMC6548564

[79] Huang Z Jiang Y Yang Y Shao J Sun X Chen J. 3,3'-Diindolylmethane alleviates oxazolone-induced colitis through Th2/Th17 suppression and Treg induction. Mol Immunol. (2013) 53:335–44. doi: 10.1016/j.molimm.2012.09.007 23085552

[80] Ehrlich AK Pennington JM Wang X Rohlman D Punj S Lohr CV. Activation of the aryl hydrocarbon receptor by 10-cl-BBQ prevents insulitis and effector T cell development independently of foxp3+ Regulatory T cells in nonobese diabetic mice. J Immunol. (2016) 196:264–73. doi: 10.4049/jimmunol.1501789 PMC4684970 26573835

[81] de Araujo EF Feriotti C Galdino NAL Preite NW Calich VLG Loures FV. The IDO-ahR axis controls th17/treg immunity in a pulmonary model of fungal infection. Front Immunol. (2017) 8:880. doi: 10.3389/fimmu.2017.00880 28791025 PMC5523665

[82] Opitz CA Litzenburger UM Sahm F Ott M Tritschler I Trump S. An endogenous tumour-promoting ligand of the human aryl hydrocarbon receptor. Nature. (2011) 478:197–203. doi: 10.1038/nature10491 21976023

[83] Bessede A Gargaro M Pallotta MT Matino D Servillo G Brunacci C. Aryl hydrocarbon receptor control of a disease tolerance defence pathway. Nature. (2014) 511:184–90. doi: 10.1038/nature13323 PMC4098076 24930766

[84] Mezrich JD Fechner JH Zhang X Johnson BP Burlingham WJ Bradfield CA. An interaction between kynurenine and the aryl hydrocarbon receptor can generate regulatory T cells. J Immunol. (2010) 185:3190–8. doi: 10.4049/jimmunol.0903670 PMC2952546 20720200

[85] Goettel JA Gandhi R Kenison JE Yeste A Murugaiyan G Sambanthamoorthy S. AHR activation is protective against colitis driven by T cells in humanized mice. Cell Rep. (2016) 17:1318–29. doi: 10.1016/j.celrep.2016.09.082 PMC5106873 27783946

[86] Tashita C Hoshi M Hirata A Nakamoto K Ando T Hattori T. Kynurenine plays an immunosuppressive role in 2,4,6-trinitrobenzene sulfate-induced colitis in mice. World J Gastroenterol. (2020) 26:918–32. doi: 10.3748/wjg.v26.i9.918 PMC7081011 32206003

[87] Gargaro M Vacca C Massari S Scalisi G Manni G Mondanelli G. Engagement of nuclear coactivator 7 by 3-hydroxyanthranilic acid enhances activation of aryl hydrocarbon receptor in immunoregulatory dendritic cells. Front Immunol. (2019) 10:1973. doi: 10.3389/fimmu.2019.01973 31481962 PMC6710348

[88] Hang S Paik D Yao L Kim E Trinath J Lu J. Bile acid metabolites control TH17 and Treg cell differentiation. Nature. (2019) 576:143–8. doi: 10.1038/s41586-019-1785-z PMC6949019 31776512

[89] Campbell C McKenney PT Konstantinovsky D Isaeva OI Schizas M Verter J. Bacterial metabolism of bile acids promotes generation of peripheral regulatory T cells. Nature. (2020) 581:475–9. doi: 10.1038/s41586-020-2193-0 PMC7540721 32461639

[90] Song X Sun X Oh SF Wu M Zhang Y Zheng W. Microbial bile acid metabolites modulate gut RORgamma(+) regulatory T cell homeostasis. Nature. (2020) 577:410–5. doi: 10.1038/s41586-019-1865-0 PMC7274525 31875848

[91] Li W Hang S Fang Y Bae S Zhang Y Zhang M. A bacterial bile acid metabolite modulates Treg activity through the nuclear hormone receptor NR4A1. Cell Host Microbe. (2021) 29:1366–77.e9. doi: 10.1016/j.chom.2021.07.013 34416161 PMC9064000

[92] Haghikia A Jorg S Duscha A Berg J Manzel A Waschbisch A. Dietary fatty acids directly impact central nervous system autoimmunity *via* the small intestine. Immunity. (2016) 44:951–3. doi: 10.1016/j.immuni.2016.04.006 27096322

[93] Furusawa Y Obata Y Fukuda S Endo TA Nakato G Takahashi D. Commensal microbe-derived butyrate induces the differentiation of colonic regulatory T cells. Nature. (2013) 504:446–50. doi: 10.1038/nature12721 24226770

[94] Smith PM Howitt MR Panikov N Michaud M Gallini CA Bohlooly YM. The microbial metabolites, short-chain fatty acids, regulate colonic Treg cell homeostasis. Science. (2013) 341:569–73. doi: 10.1126/science.1241165 PMC3807819 23828891

[95] Galeano Nino JL Wu H LaCourse KD Kempchinsky AG Baryiames A Barber B. Effect of the intratumoral microbiota on spatial and cellular heterogeneity in cancer. Nature. (2022) 611:810–7. doi: 10.1038/s41586-022-05435-0 PMC9684076 36385528

[96] Zhang Q Ma C Duan Y Heinrich B Rosato U Diggs LP. Gut microbiome directs hepatocytes to recruit MDSCs and promote cholangiocarcinoma. Cancer Discovery. (2021) 11:1248–67. doi: 10.1158/2159-8290.CD-20-0304 PMC8102309 33323397

[97] Chang S Kim YH Kim YJ Kim YW Moon S Lee YY. Taurodeoxycholate increases the number of myeloid-derived suppressor cells that ameliorate sepsis in mice. Front Immunol. (2018) 9:1984. doi: 10.3389/fimmu.2018.01984 30279688 PMC6153344

[98] Neamah WH Busbee PB Alghetaa H Abdulla OA Nagarkatti M Nagarkatti P. AhR activation leads to alterations in the gut microbiome with consequent effect on induction of myeloid derived suppressor cells in a CXCR2-dependent manner. Int J Mol Sci. (2020) 21:9613. doi: 10.3390/ijms21249613 33348596 PMC7767008

[99] Mascanfroni ID Takenaka MC Yeste A Patel B Wu Y Kenison JE. Metabolic control of type 1 regulatory T cell differentiation by AHR and HIF1-alpha. Nat Med. (2015) 21:638–46. doi: 10.1038/nm.3868 PMC4476246 26005855

[100] Apetoh L Quintana FJ Pot C Joller N Xiao S Kumar D. The aryl hydrocarbon receptor interacts with c-Maf to promote the differentiation of type 1 regulatory T cells induced by IL-27. Nat Immunol. (2010) 11:854–61. doi: 10.1038/ni.1912 PMC2940320 20676095

[101] Piper CJM Rosser EC Oleinika K Nistala K Krausgruber T Rendeiro AF. Aryl hydrocarbon receptor contributes to the transcriptional program of IL-10-producing regulatory B cells. Cell Rep. (2019) 29:1878–92.e7. doi: 10.1016/j.celrep.2019.10.018 31722204 PMC6856759

[102] Su X Zhang M Qi H Gao Y Yang Y Yun H. Gut microbiota-derived metabolite 3-idoleacetic acid together with LPS induces IL-35(+) B cell generation. Microbiome. (2022) 10:13. doi: 10.1186/s40168-021-01205-8 35074011 PMC8785567

[103] Shen J Yang L You K Chen T Su Z Cui Z. Indole-3-acetic acid alters intestinal microbiota and alleviates ankylosing spondylitis in mice. Front Immunol. (2022) 13:762580. doi: 10.3389/fimmu.2022.762580 35185872 PMC8854167

[104] Gutierrez-Vazquez C Quintana FJ. Regulation of the immune response by the aryl hydrocarbon receptor. Immunity. (2018) 48:19–33. doi: 10.1016/j.immuni.2017.12.012 29343438 PMC5777317

[105] Zelante T Iannitti RG Cunha C De Luca A Giovannini G Pieraccini G. Tryptophan catabolites from microbiota engage aryl hydrocarbon receptor and balance mucosal reactivity *via* interleukin-22. Immunity. (2013) 39:372–85. doi: 10.1016/j.immuni.2013.08.003 23973224

[106] Lee JS Cella M McDonald KG Garlanda C Kennedy GD Nukaya M. AHR drives the development of gut ILC22 cells and postnatal lymphoid tissues *via* pathways dependent on and independent of Notch. Nat Immunol. (2011) 13:144–51. doi: 10.1038/ni.2187 PMC3468413 22101730

[107] Qiu J Heller JJ Guo X Chen ZM Fish K Fu YX. The aryl hydrocarbon receptor regulates gut immunity through modulation of innate lymphoid cells. Immunity. (2012) 36:92–104. doi: 10.1016/j.immuni.2011.11.011 22177117 PMC3268875

[108] Ebihara T Song C Ryu SH Plougastel-Douglas B Yang L Levanon D. Runx3 specifies lineage commitment of innate lymphoid cells. Nat Immunol. (2015) 16:1124–33. doi: 10.1038/ni.3272 PMC4618046 26414766

[109] Veldhoen M Hirota K Westendorf AM Buer J Dumoutier L Renauld JC. The aryl hydrocarbon receptor links TH17-cell-mediated autoimmunity to environmental toxins. Nature. (2008) 453:106–9. doi: 10.1038/nature06881 18362914

[110] Lanz TV Becker S Mohapatra SR Opitz CA Wick W Platten M. Suppression of Th1 differentiation by tryptophan supplementation in *vivo* . Amino Acids. (2017) 49:1169–75. doi: 10.1007/s00726-017-2415-4 28421297

[111] Ding C Hong Y Che Y He T Wang Y Zhang S. Bile acid restrained T cell activation explains cholestasis aggravated hepatitis B virus infection. FASEB J. (2022) 36:e22468. doi: 10.1096/fj.202200332R 35913801

[112] Cao W Kayama H Chen ML Delmas A Sun A Kim SY. The xenobiotic transporter mdr1 enforces T cell homeostasis in the presence of intestinal bile acids. Immunity. (2017) 47:1182–96.e10. doi: 10.1016/j.immuni.2017.11.012 29262351 PMC5741099

[113] Pols TWH Puchner T Korkmaz HI Vos M Soeters MR de Vries CJM. Lithocholic acid controls adaptive immune responses by inhibition of Th1 activation through the Vitamin D receptor. PloS One. (2017) 12:e0176715. doi: 10.1371/journal.pone.0176715 28493883 PMC5426628

[114] Ivanov II McKenzie BS Zhou L Tadokoro CE Lepelley A Lafaille JJ. The orphan nuclear receptor RORgammat directs the differentiation program of proinflammatory IL-17+ T helper cells. Cell. (2006) 126:1121–33. doi: 10.1016/j.cell.2006.07.035 16990136

[115] Chung DJ Rossi M Romano E Ghith J Yuan J Munn DH. Indoleamine 2,3-dioxygenase-expressing mature human monocyte-derived dendritic cells expand potent autologous regulatory T cells. Blood. (2009) 114:555–63. doi: 10.1182/blood-2008-11-191197 PMC2713474 19465693

[116] Trompette A Gollwitzer ES Yadava K Sichelstiel AK Sprenger N Ngom-Bru C. Gut microbiota metabolism of dietary fiber influences allergic airway disease and hematopoiesis. Nat Med. (2014) 20:159–66. doi: 10.1038/nm.3444 24390308

[117] Rosser EC Piper CJM Matei DE Blair PA Rendeiro AF Orford M. Microbiota-derived metabolites suppress arthritis by amplifying aryl-hydrocarbon receptor activation in regulatory B cells. Cell Metab. (2020) 31:837–51.e10. doi: 10.1016/j.cmet.2020.03.003 32213346 PMC7156916

[118] Yao Y Cai X Zheng Y Zhang M Fei W Sun D. Short-chain fatty acids regulate B cells differentiation *via* the FFA2 receptor to alleviate rheumatoid arthritis. Br J Pharmacol. (2022) 179:4315–29. doi: 10.1111/bph.15852 35393660

[119] Kibbie JJ Dillon SM Thompson TA Purba CM McCarter MD Wilson CC. Butyrate directly decreases human gut lamina propria CD4 T cell function through histone deacetylase (HDAC) inhibition and GPR43 signaling. Immunobiology. (2021) 226:152126. doi: 10.1016/j.imbio.2021.152126 34365090 PMC8478853

[120] Gury-BenAri M Thaiss CA Serafini N Winter DR Giladi A Lara-Astiaso D. The spectrum and regulatory landscape of intestinal innate lymphoid cells are shaped by the microbiome. Cell. (2016) 166:1231–46 e13. doi: 10.1016/j.cell.2016.07.043 27545347

[121] Han JX Tao ZH Wang JL Zhang L Yu CY Kang ZR. Microbiota-derived tryptophan catabolites mediate the chemopreventive effects of statins on colorectal cancer. Nat Microbiol. (2023) 8:919–33. doi: 10.1038/s41564-023-01363-5 37069401

[122] Liu J Wei Y Jia W Can C Wang R Yang X. Chenodeoxycholic acid suppresses AML progression through promoting lipid peroxidation *via* ROS/p38 MAPK/DGAT1 pathway and inhibiting M2 macrophage polarization. Redox Biol. (2022) 56:102452. doi: 10.1016/j.redox.2022.102452 36084349 PMC9465103

[123] Al-Qadami GH Secombe KR Subramaniam CB Wardill HR Bowen JM. Gut microbiota-derived short-chain fatty acids: impact on cancer treatment response and toxicities. Microorganisms. (2022) 10:2048. doi: 10.3390/microorganisms10102048 36296324 PMC9612155

[124] He Y Fu L Li Y Wang W Gong M Zhang J. Gut microbial metabolites facilitate anticancer therapy efficacy by modulating cytotoxic CD8(+) T cell immunity. Cell Metab. (2021) 33:988–1000.e7. doi: 10.1016/j.cmet.2021.03.002 33761313

[125] Siddiqui I Schaeuble K Chennupati V Fuertes Marraco SA Calderon-Copete S Pais Ferreira D. Intratumoral tcf1(+)PD-1(+)CD8(+) T cells with stem-like properties promote tumor control in response to vaccination and checkpoint blockade immunotherapy. Immunity. (2019) 50:195–211.e10. doi: 10.1016/j.immuni.2018.12.021 30635237

[126] Lam KC Araya RE Huang A Chen Q Di Modica M Rodrigues RR. Microbiota triggers STING-type I IFN-dependent monocyte reprogramming of the tumor microenvironment. Cell. (2021) 184:5338–56.e21. doi: 10.1016/j.cell.2021.09.019 34624222 PMC8650838

[127] Wang H Rong X Zhao G Zhou Y Xiao Y Ma D. The microbial metabolite trimethylamine N-oxide promotes antitumor immunity in triple-negative breast cancer. Cell Metab. (2022) 34:581–94.e8. doi: 10.1016/j.cmet.2022.02.010 35278352

[128] Mager LF Burkhard R Pett N Cooke NCA Brown K Ramay H. Microbiome-derived inosine modulates response to checkpoint inhibitor immunotherapy. Science. (2020) 369:1481–9. doi: 10.1126/science.abc3421 32792462

[129] Vernocchi P Gili T Conte F Del Chierico F Conta G Miccheli A. Network analysis of gut microbiome and metabolome to discover microbiota-linked biomarkers in patients affected by non-small cell lung cancer. Int J Mol Sci. (2020) 21:8730. doi: 10.3390/ijms21228730 33227982 PMC7699235

[130] Gopalakrishnan V Spencer CN Nezi L Reuben A Andrews MC Karpinets TV. Gut microbiome modulates response to anti-PD-1 immunotherapy in melanoma patients. Science. (2018) 359:97–103. doi: 10.1126/science.aan4236 29097493 PMC5827966

[131] Routy B Le Chatelier E Derosa L Duong CPM Alou MT Daillere R. Gut microbiome influences efficacy of PD-1-based immunotherapy against epithelial tumors. Science. (2018) 359:91–7. doi: 10.1126/science.aan3706 29097494

[132] Sivan A Corrales L Hubert N Williams JB Aquino-Michaels K Earley ZM. Commensal Bifidobacterium promotes antitumor immunity and facilitates anti-PD-L1 efficacy. Science. (2015) 350:1084–9. doi: 10.1126/science.aac4255 PMC4873287 26541606

[133] Deng H Li Z Tan Y Guo Z Liu Y Wang Y. A novel strain of Bacteroides fragilis enhances phagocytosis and polarises M1 macrophages. Sci Rep. (2016) 6:29401. doi: 10.1038/srep29401 27381366 PMC4933912

[134] Mollazadeh H Cicero AFG Blesso CN Pirro M Majeed M Sahebkar A. Immune modulation by curcumin: The role of interleukin-10. Crit Rev Food Sci Nutr. (2019) 59:89–101. doi: 10.1080/10408398.2017.1358139 28799796

[135] Colombo MP Piconese S. Regulatory-T-cell inhibition versus depletion: the right choice in cancer immunotherapy. Nat Rev Cancer. (2007) 7:880–7. doi: 10.1038/nrc2250 17957190

[136] Tay C Tanaka A Sakaguchi S. Tumor-infiltrating regulatory T cells as targets of cancer immunotherapy. Cancer Cell. (2023) 41:450–65. doi: 10.1016/j.ccell.2023.02.014 36917950

[137] Sawant DV Yano H Chikina M Zhang Q Liao M Liu C. Adaptive plasticity of IL-10(+) and IL-35(+) T(reg) cells cooperatively promotes tumor T cell exhaustion. Nat Immunol. (2019) 20:724–35. doi: 10.1038/s41590-019-0346-9 PMC6531353 30936494

[138] Tanaka A Sakaguchi S. Regulatory T cells in cancer immunotherapy. Cell Res. (2017) 27:109–18. doi: 10.1038/cr.2016.151 PMC5223231 27995907

[139] Maj T Wang W Crespo J Zhang H Wang W Wei S. Oxidative stress controls regulatory T cell apoptosis and suppressor activity and PD-L1-blockade resistance in tumor. Nat Immunol. (2017) 18:1332–41. doi: 10.1038/ni.3868 PMC5770150 29083399

[140] Chimote AA Balajthy A Arnold MJ Newton HS Hajdu P Qualtieri J. A defect in KCa3.1 channel activity limits the ability of CD8(+) T cells from cancer patients to infiltrate an adenosine-rich microenvironment. Sci Signal. (2018) 11:eaaq1616. doi: 10.1126/scisignal.aaq1616 29692361 PMC6006512

[141] Vijayan D Young A Teng MWL Smyth MJ. Targeting immunosuppressive adenosine in cancer. Nat Rev Cancer. (2017) 17:709–24. doi: 10.1038/nrc.2017.86 29059149

[142] Whiteside TL Jackson EK. Adenosine and prostaglandin e2 production by human inducible regulatory T cells in health and disease. Front Immunol. (2013) 4:212. doi: 10.3389/fimmu.2013.00212 23898333 PMC3722515

[143] Nagaraj S Gupta K Pisarev V Kinarsky L Sherman S Kang L. Altered recognition of antigen is a mechanism of CD8+ T cell tolerance in cancer. Nat Med. (2007) 13:828–35. doi: 10.1038/nm1609 PMC2135607 17603493

[144] Grossman WJ Verbsky JW Barchet W Colonna M Atkinson JP Ley TJ. Human T regulatory cells can use the perforin pathway to cause autologous target cell death. Immunity. (2004) 21:589–601. doi: 10.1016/j.immuni.2004.09.002 15485635

[145] Chen D Xie J Fiskesund R Dong W Liang X Lv J. Chloroquine modulates antitumor immune response by resetting tumor-associated macrophages toward M1 phenotype. Nat Commun. (2018) 9:873. doi: 10.1038/s41467-018-03225-9 29491374 PMC5830447

[146] Qu QX Huang Q Shen Y Zhu YB Zhang XG. The increase of circulating PD-L1-expressing CD68(+) macrophage in ovarian cancer. Tumour Biol. (2016) 37:5031–7. doi: 10.1007/s13277-015-4066-y 26541760

[147] Zhu Z Zhang H Chen B Liu X Zhang S Zong Z. PD-L1-mediated immunosuppression in glioblastoma is associated with the infiltration and M2-polarization of tumor-associated macrophages. Front Immunol. (2020) 11:588552. doi: 10.3389/fimmu.2020.588552 33329573 PMC7734279

[148] Tan B Shi X Zhang J Qin J Zhang N Ren H. Inhibition of rspo-lgr4 facilitates checkpoint blockade therapy by switching macrophage polarization. Cancer Res. (2018) 78:4929–42. doi: 10.1158/0008-5472.CAN-18-0152 29967265

[149] Farhood B Khodamoradi E Hoseini-Ghahfarokhi M Motevaseli E Mirtavoos-Mahyari H Eleojo Musa A. TGF-beta in radiotherapy: Mechanisms of tumor resistance and normal tissues injury. Pharmacol Res. (2020) 155:104745. doi: 10.1016/j.phrs.2020.104745 32145401

[150] Salminen A. Immunosuppressive network promotes immunosenescence associated with aging and chronic inflammatory conditions. J Mol Med (Berl). (2021) 99:1553–69. doi: 10.1007/s00109-021-02123-w PMC8384586 34432073

[151] Cui W Wang Z Lv J Qin Y Shi H. MDSCs: the key players in the formation of pre-metastatic niche. Front Biosci (Landmark Ed). (2023) 28:58. doi: 10.31083/j.fbl2803058 37005751

[152] Zhou X Fang D Liu H Ou X Zhang C Zhao Z. PMN-MDSCs accumulation induced by CXCL1 promotes CD8(+) T cells exhaustion in gastric cancer. Cancer Lett. (2022) 532:215598. doi: 10.1016/j.canlet.2022.215598 35176418

[153] Mao FY Zhao YL Lv YP Teng YS Kong H Liu YG. CD45(+)CD33(low)CD11b(dim) myeloid-derived suppressor cells suppress CD8(+) T cell activity *via* the IL-6/IL-8-arginase I axis in human gastric cancer. Cell Death Dis. (2018) 9:763. doi: 10.1038/s41419-018-0803-7 29988030 PMC6037756

[154] Gibson JT Orlandella RM Turbitt WJ Behring M Manne U Sorge RE. Obesity-associated myeloid-derived suppressor cells promote apoptosis of tumor-infiltrating CD8 T cells and immunotherapy resistance in breast cancer. Front Immunol. (2020) 11:590794. doi: 10.3389/fimmu.2020.590794 33123173 PMC7573510

[155] Tong L Jimenez-Cortegana C Tay AHM Wickstrom S Galluzzi L Lundqvist A. NK cells and solid tumors: therapeutic potential and persisting obstacles. Mol Cancer. (2022) 21:206. doi: 10.1186/s12943-022-01672-z 36319998 PMC9623927

[156] Sarhan D Hippen KL Lemire A Hying S Luo X Lenvik T. Adaptive NK cells resist regulatory T-cell suppression driven by IL37. Cancer Immunol Res. (2018) 6:766–75. doi: 10.1158/2326-6066.CIR-17-0498 PMC6030483 29784636

[157] Zimmer J Andres E Hentges F. NK cells and Treg cells: a fascinating dance cheek to cheek. Eur J Immunol. (2008) 38:2942–5. doi: 10.1002/eji.200838813 18979513

[158] Eisinger S Sarhan D Boura VF Ibarlucea-Benitez I Tyystjarvi S Oliynyk G. Targeting a scavenger receptor on tumor-associated macrophages activates tumor cell killing by natural killer cells. Proc Natl Acad Sci U S A. (2020) 117:32005–16. doi: 10.1073/pnas.2015343117 PMC7750482 33229588

[159] Greene S Robbins Y Mydlarz WK Huynh AP Schmitt NC Friedman J. Inhibition of MDSC trafficking with SX-682, a CXCR1/2 inhibitor, enhances NK-cell immunotherapy in head and neck cancer models. Clin Cancer Res. (2020) 26:1420–31. doi: 10.1158/1078-0432.CCR-19-2625 PMC7073293 31848188

[160] Li Z Pang Y Gara SK Achyut BR Heger C Goldsmith PK. Gr-1+CD11b+ cells are responsible for tumor promoting effect of TGF-beta in breast cancer progression. Int J Cancer. (2012) 131:2584–95. doi: 10.1002/ijc.27572 PMC3433574 22487809

[161] Veglia F Sanseviero E Gabrilovich DI. Myeloid-derived suppressor cells in the era of increasing myeloid cell diversity. Nat Rev Immunol. (2021) 21:485–98. doi: 10.1038/s41577-020-00490-y PMC7849958 33526920

[162] Ou Y Cannon MJ Nakagawa M. Regulatory T cells in gynecologic cancer. MOJ Immunol. (2018) 6:34–42. 30637330 PMC6329475

[163] Gur C Ibrahim Y Isaacson B Yamin R Abed J Gamliel M. Binding of the Fap2 protein of Fusobacterium nucleatum to human inhibitory receptor TIGIT protects tumors from immune cell attack. Immunity. (2015) 42:344–55. doi: 10.1016/j.immuni.2015.01.010 PMC4361732 25680274

[164] Yu Q Newsome RC Beveridge M Hernandez MC Gharaibeh RZ Jobin C. Intestinal microbiota modulates pancreatic carcinogenesis through intratumoral natural killer cells. Gut Microbes. (2022) 14:2112881. doi: 10.1080/19490976.2022.2112881 35980869 PMC9397420

[165] Hezaveh K Shinde RS Klotgen A Halaby MJ Lamorte S Ciudad MT. Tryptophan-derived microbial metabolites activate the aryl hydrocarbon receptor in tumor-associated macrophages to suppress anti-tumor immunity. Immunity. (2022) 55:324–40 e8. doi: 10.1016/j.immuni.2022.01.006 35139353 PMC8888129

[166] Campesato LF Budhu S Tchaicha J Weng CH Gigoux M Cohen IJ. Blockade of the AHR restricts a Treg-macrophage suppressive axis induced by L-Kynurenine. Nat Commun. (2020) 11:4011. doi: 10.1038/s41467-020-17750-z 32782249 PMC7419300

[167] Liu Y Zhou N Zhou L Wang J Zhou Y Zhang T. IL-2 regulates tumor-reactive CD8(+) T cell exhaustion by activating the aryl hydrocarbon receptor. Nat Immunol. (2021) 22:358–69. doi: 10.1038/s41590-020-00850-9 33432230

[168] Fallarino F Grohmann U Vacca C Bianchi R Orabona C Spreca A. T cell apoptosis by tryptophan catabolism. Cell Death Differ. (2002) 9:1069–77. doi: 10.1038/sj.cdd.4401073 12232795

[169] Frumento G Rotondo R Tonetti M Damonte G Benatti U Ferrara GB. Tryptophan-derived catabolites are responsible for inhibition of T and natural killer cell proliferation induced by indoleamine 2,3-dioxygenase. J Exp Med. (2002) 196:459–68. doi: 10.1084/jem.20020121 PMC2196046 12186838

[170] Song H Park H Kim YS Kim KD Lee HK Cho DH. L-kynurenine-induced apoptosis in human NK cells is mediated by reactive oxygen species. Int Immunopharmacol. (2011) 11:932–8. doi: 10.1016/j.intimp.2011.02.005 21352963

[171] Kassayova M Bobrov N Strojny L Orendas P Demeckova V Jendzelovsky R. Anticancer and immunomodulatory effects of lactobacillus plantarum LS/07, inulin and melatonin in NMU-induced rat model of breast cancer. Anticancer Res. (2016) 36:2719–28. 27272781

[172] Lan X Ma J Huang Z Xu Y Hu Y. Akkermansia muciniphila might improve anti-PD-1 therapy against HCC by changing host bile acid metabolism. J Gene Med. (2024) 26:e3639. doi: 10.1002/jgm.3639 38058259

[173] Bender MJ McPherson AC Phelps CM Pandey SP Laughlin CR Shapira JH. Dietary tryptophan metabolite released by intratumoral Lactobacillus reuteri facilitates immune checkpoint inhibitor treatment. Cell. (2023) 186:1846–62 e26. doi: 10.1016/j.cell.2023.03.011 37028428 PMC10148916

[174] Xue L Wang C Qian Y Zhu W Liu L Yang X. Tryptophan metabolism regulates inflammatory macrophage polarization as a predictive factor for breast cancer immunotherapy. Int Immunopharmacol. (2023) 125:111196. doi: 10.1016/j.intimp.2023.111196 37972471

[175] Yu X Ou J Wang L Li Z Ren Y Xie L. Gut microbiota modulate CD8(+) T cell immunity in gastric cancer through Butyrate/GPR109A/HOPX. Gut Microbes. (2024) 16:2307542. doi: 10.1080/19490976.2024.2307542 38319728 PMC10854374

[176] Majoros A Platanitis E Kernbauer-Holzl E Rosebrock F Muller M Decker T. Canonical and non-canonical aspects of JAK-STAT signaling: lessons from interferons for cytokine responses. Front Immunol. (2017) 8:29. doi: 10.3389/fimmu.2017.00029 28184222 PMC5266721

[177] Liang YH Chen KH Tsai JH Cheng YM Lee CC Kao CH. Proteasome inhibitors restore the STAT1 pathway and enhance the expression of MHC class I on human colon cancer cells. J BioMed Sci. (2021) 28:75. doi: 10.1186/s12929-021-00769-9 34758826 PMC8579664

[178] Coupel S Moreau A Hamidou M Horejsi V Soulillou JP Charreau B. Expression and release of soluble HLA-E is an immunoregulatory feature of endothelial cell activation. Blood. (2007) 109:2806–14. doi: 10.1182/blood-2006-06-030213 17179229

[179] Pereira BI Devine OP Vukmanovic-Stejic M Chambers ES Subramanian P Patel N. Senescent cells evade immune clearance *via* HLA-E-mediated NK and CD8(+) T cell inhibition. Nat Commun. (2019) 10:2387. doi: 10.1038/s41467-019-10335-5 31160572 PMC6547655

[180] Sheehy MJ Meske LM Emler CA Rowe JR Neme de Gimenez MH Ingle CA. Allelic T-cell receptor alpha complexes have little or no influence on susceptibility to type 1 diabetes. Hum Immunol. (1989) 26:261–71. doi: 10.1016/0198-8859(89)90004-9 2573589

[181] Lin Z Bashirova AA Viard M Garner L Quastel M Beiersdorfer M. HLA class I signal peptide polymorphism determines the level of CD94/NKG2-HLA-E-mediated regulation of effector cell responses. Nat Immunol. (2023) 24:1087–97. doi: 10.1038/s41590-023-01523-z PMC10690437 37264229

[182] Ducoin K Oger R Bilonda Mutala L Deleine C Jouand N Desfrancois J. Targeting NKG2A to boost anti-tumor CD8 T-cell responses in human colorectal cancer. Oncoimmunology. (2022) 11:2046931. doi: 10.1080/2162402X.2022.2046931 35295095 PMC8920231

[183] Sun G Zhao X Li M Zhang C Jin H Li C. CD4 derived double negative T cells prevent the development and progression of nonalcoholic steatohepatitis. Nat Commun. (2021) 12:650. doi: 10.1038/s41467-021-20941-x 33510172 PMC7844244

[184] Huang JJ Blobe GC. Dichotomous roles of TGF-beta in human cancer. Biochem Soc Trans. (2016) 44:1441–54. doi: 10.1042/BST20160065 PMC5682628 27911726

[185] Mingari MC Vitale C Cantoni C Bellomo R Ponte M Schiavetti F. Interleukin-15-induced maturation of human natural killer cells from early thymic precursors: selective expression of CD94/NKG2-A as the only HLA class I-specific inhibitory receptor. Eur J Immunol. (1997) 27:1374–80. doi: 10.1002/eji.1830270612 9209487

[186] Sheu BC Chiou SH Lin HH Chow SN Huang SC Ho HN. Up-regulation of inhibitory natural killer receptors CD94/NKG2A with suppressed intracellular perforin expression of tumor-infiltrating CD8+ T lymphocytes in human cervical carcinoma. Cancer Res. (2005) 65:2921–9. doi: 10.1158/0008-5472.CAN-04-2108 15805295

[187] Braud VM Aldemir H Breart B Ferlin WG. Expression of CD94-NKG2A inhibitory receptor is restricted to a subset of CD8+ T cells. Trends Immunol. (2003) 24:162–4. doi: 10.1016/s1471-4906(03)00064-4 12697440

[188] Bertone S Schiavetti F Bellomo R Vitale C Ponte M Moretta L. Transforming growth factor-beta-induced expression of CD94/NKG2A inhibitory receptors in human T lymphocytes. Eur J Immunol. (1999) 29:23–9. doi: 10.1002/(SICI)1521-4141(199901)29:01<23::AID-IMMU23>3.0.CO;2-Y 9933082

[189] Jabri B Selby JM Negulescu H Lee L Roberts AI Beavis A. TCR specificity dictates CD94/NKG2A expression by human CTL. Immunity. (2002) 17:487–99. doi: 10.1016/s1074-7613(02)00427-2 12387742

[190] Mingari MC Ponte M Bertone S Schiavetti F Vitale C Bellomo R. HLA class I-specific inhibitory receptors in human T lymphocytes: interleukin 15-induced expression of CD94/NKG2A in superantigen- or alloantigen-activated CD8+ T cells. Proc Natl Acad Sci U S A. (1998) 95:1172–7. doi: 10.1073/pnas.95.3.1172 PMC18710 9448304

[191] Zhang Y Zhang Z. The history and advances in cancer immunotherapy: understanding the characteristics of tumor-infiltrating immune cells and their therapeutic implications. Cell Mol Immunol. (2020) 17:807–21. doi: 10.1038/s41423-020-0488-6 PMC7395159 32612154

[192] Matson V Fessler J Bao R Chongsuwat T Zha Y Alegre ML. The commensal microbiome is associated with anti-PD-1 efficacy in metastatic melanoma patients. Science. (2018) 359:104–8. doi: 10.1126/science.aao3290 PMC6707353 29302014

[193] Fazzino L Anisman J Chacon JM Harcombe WR. Phage cocktail strategies for the suppression of a pathogen in a cross-feeding coculture. Microb Biotechnol. (2020) 13:1997–2007. doi: 10.1111/1751-7915.13650 32814365 PMC7533344

[194] Wong CC Yu J. Gut microbiota in colorectal cancer development and therapy. Nat Rev Clin Oncol. (2023) 20:429–52. doi: 10.1038/s41571-023-00766-x 37169888

[195] Lee SH Cho SY Yoon Y Park C Sohn J Jeong JJ. Bifidobacterium bifidum strains synergize with immune checkpoint inhibitors to reduce tumour burden in mice. Nat Microbiol. (2021) 6:277–88. doi: 10.1038/s41564-020-00831-6 33432149

[196] Montalban-Arques A Katkeviciute E Busenhart P Bircher A Wirbel J Zeller G. Commensal Clostridiales strains mediate effective anti-cancer immune response against solid tumors. Cell Host Microbe. (2021) 29:1573–88 e7. doi: 10.1016/j.chom.2021.08.001 34453895

[197] Lu Y Yuan X Wang M He Z Li H Wang J. Gut microbiota influence immunotherapy responses: mechanisms and therapeutic strategies. J Hematol Oncol. (2022) 15:47. doi: 10.1186/s13045-022-01273-9 35488243 PMC9052532

[198] Derosa L Routy B Fidelle M Iebba V Alla L Pasolli E. Gut bacteria composition drives primary resistance to cancer immunotherapy in renal cell carcinoma patients. Eur Urol. (2020) 78:195–206. doi: 10.1016/j.eururo.2020.04.044 32376136

